# A Novel 7*H*-[1,2,4]Triazolo[3,4-*b*]thiadiazine-based Cystic Fibrosis Transmembrane Conductance Regulator Potentiator Directed toward Treatment of Cystic Fibrosis

**DOI:** 10.1021/acsmedchemlett.3c00155

**Published:** 2023-09-20

**Authors:** Andras Rab, Xun Yang, William F. Tracy, Jeong S. Hong, Disha Joshi, Candela Manfredi, Sadhana S. Ponnaluri, Alexander A. Kolykhalov, Min Qui, Haian Fu, Yuhong Du, Huw M. L. Davies, Eric J. Sorscher

**Affiliations:** † Department of Pediatrics, Emory University School of Medicine, Atlanta, Georgia 30322, United States; ‡ Department of Chemistry, Emory University, 1515 Dickey Dr., Atlanta, Georgia 30329, United States; § Department of Pharmacology and Chemical Biology, Emory University School of Medicine, Atlanta, Georgia 30322, United States; ⊥ Emory Chemical Biology Discovery Center, Emory University School of Medicine, Atlanta, Georgia 30322, United States; ∥ Emory Institute for Drug Development, Atlanta, Georgia 30322, United States

**Keywords:** CFTR, high throughput compound library screening, drug discovery, cystic fibrosis

## Abstract

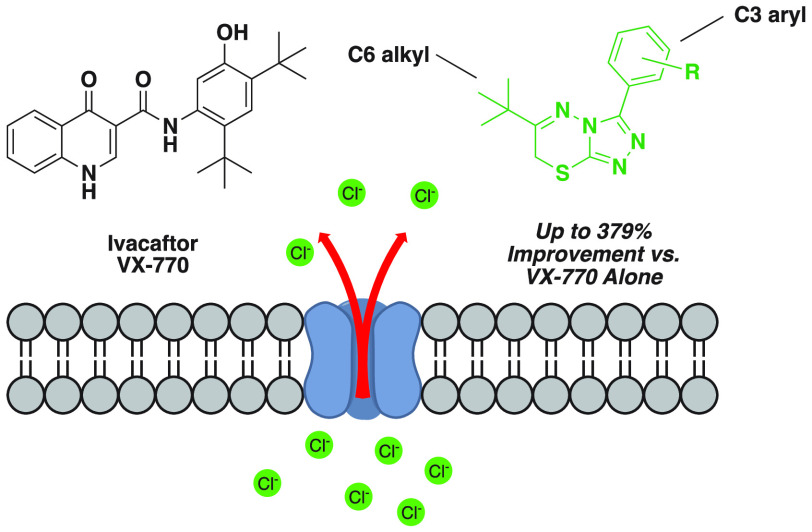

Cystic fibrosis (CF) is an autosomal genetic disorder caused by disrupted anion transport in epithelial cells lining tissues in the human airways and digestive system. While cystic fibrosis transmembrane conductance regulator (CFTR) modulator compounds have provided transformative improvement in CF respiratory function, certain patients exhibit marginal clinical benefit or detrimental effects or have a form of the disease not approved or unlikely to respond using CFTR modulation. We tested hit compounds from a 300,000-drug screen for their ability to augment CFTR transepithelial transport alone or in combination with the FDA-approved CFTR potentiator ivacaftor (VX-770). A subsequent SAR campaign led us to a class of 7*H*-[1,2,4]triazolo[3,4-*b*][1,3,4]thiadiazines that in combination with VX-770 rescued function of G551D mutant CFTR channels to approximately 400% above the activity of VX-770 alone and to nearly wild-type CFTR levels in the same Fischer rat thyroid model system.

Cystic fibrosis (CF) is a congenital disorder caused by impaired function of the cystic fibrosis transmembrane conductance regulator (CFTR) protein,^1^ which functions as an epithelial anion channel. The disorder affects over 80,000 patients globally,^2^ and >2,000 mutations have been identified in the human gene with approximately 400 of these well established as causing CF.^3^ Impairment of CFTR manifests as a multiorgan disease primarily disrupting respiratory, gastrointestinal, reproductive, and other exocrine tissues. Pulmonary manifestations comprise the major cause of morbidity and mortality, with blockage of airways due to thick and viscous mucous, chronic superinfection (*Pseudomonas aeruginosa* and multiple other bacterial pathogens), pulmonary inflammation,^4^ and structural consequences that include bronchiectasis, and parenchymal fibrosis.^5^ High throughput compound library screening has identified novel activators of mutant CFTR that have recently become part of standard care.^5^ These include CFTR “potentiators” (which stimulate gating/ion transport through CFTR) and “correctors” (which augment steady-state levels of CFTR protein at the plasma membrane).^6^

Fischer rat thyroid (FRT) cell lines expressing recombinant CFTR have become a workhorse for cystic fibrosis drug discovery and research, including compound library screening,^7−9^ optimization, and pharmaceutical submissions for regulatory approval.^10^ We utilized these cells in high throughput format to identify small molecule candidates that improve the trafficking and/or function of mutant CFTR, and we tested SAR to optimize a lead compound series. Our approach resulted in the development of novel CFTR modulators exhibiting substantial CFTR rescue as part of combination treatments with the FDA-approved compound VX-770. Examples of modulators currently available for CF care are shown in Figure 1.^11−15^

**Figure 1:**
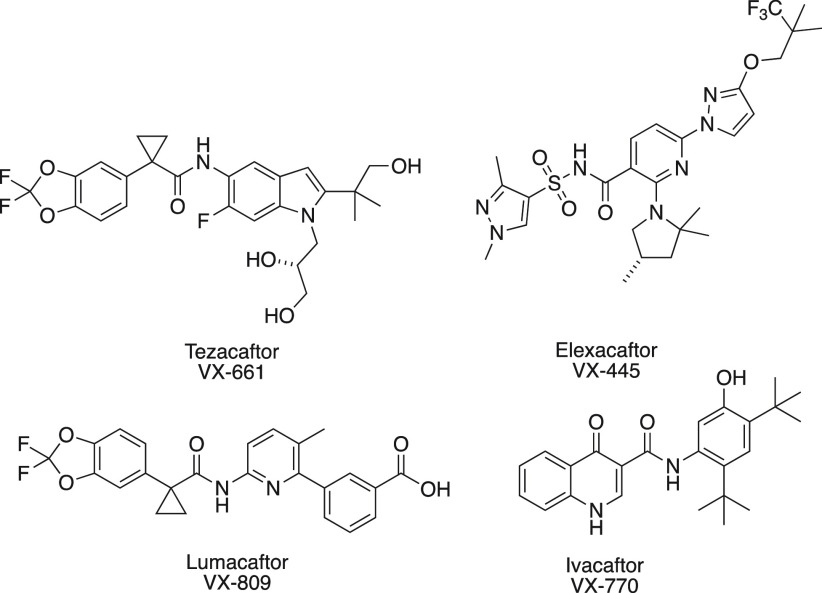
FDA-approved CFTR modulators.

G551D is the second most common CF mutation in the United States (observed in 4%–5% of the CF patient population^11^) and the prototype variant for which the CFTR potentiator VX-770 (Figure 1) was originally approved. VX-770 enhances G551D CFTR chloride channel function by directly binding the mutant protein and augmenting channel open probability.^10,16,17^ Our objective in the current study was to identify compounds that enhanced the activity of VX-770 against a variety of mutant CFTRs. For this purpose, a ∼300,000 compound library was screened against a relatively common CFTR defect (N1303K) for which modulator therapy is not yet available. We identified compounds that modestly enhance CFTR abundance at the cell surface and also confer strong co-potentiation of numerous CFTRs, including G551D. Dual-acting agents of this type have been described previously^18−20^ and in some cases bind CFTR to promote folding in a manner that augments both maturational processing and gating, thereby increasing transepithelial chloride transport. Another mechanism that may confer dual activity involves drugs that enhance CFTR insertion at the cell surface (e.g., from a sub-plasma-membrane compartment) and increase CFTR-dependent ion transport.^21^ In the present study, following structure–activity optimization, we show a surprising and robust level of activation mediated by the new compound class when assessed in combination with VX-770.

Our primary chemical screen utilized a library of 300,000 compounds and was performed on FRT cells stably expressing N1303K CFTR encoding a horseradish peroxidase (HRP) tag in the fourth extracellular protein loop. This configuration allowed detection of CFTR at the plasma membrane by a cell-based enzyme-linked immunoassay (ELISA) to monitor HRP activity. The hit compounds **1**–**3** (Figure 2) were identified by virtue of modest stabilization of surface N1303K CFTR in this fashion. Supplemental Figure 1 provides an example of HRP-tagged N1303K CFTR enriched in the plasma membrane following treatment with compound **3** as judged by cell-based HRP ELISA. A significant attribute of the confirmed hit compounds surfaced when they were tested functionally on CF-causing mutations such as N1303K CFTR (Supplemental Figure 2). Compound **3** significantly enhances N1303K activity when combined with the elexacaftor/tezacaftor/ivacaftor (ETI) treatment. The N1303K variant has not been approved for CFTR modulator therapy, and our FRT data indicate substantial improvement of function to a level predictive of clinical benefit (Supplemental Figure 2).

**Figure 2:**
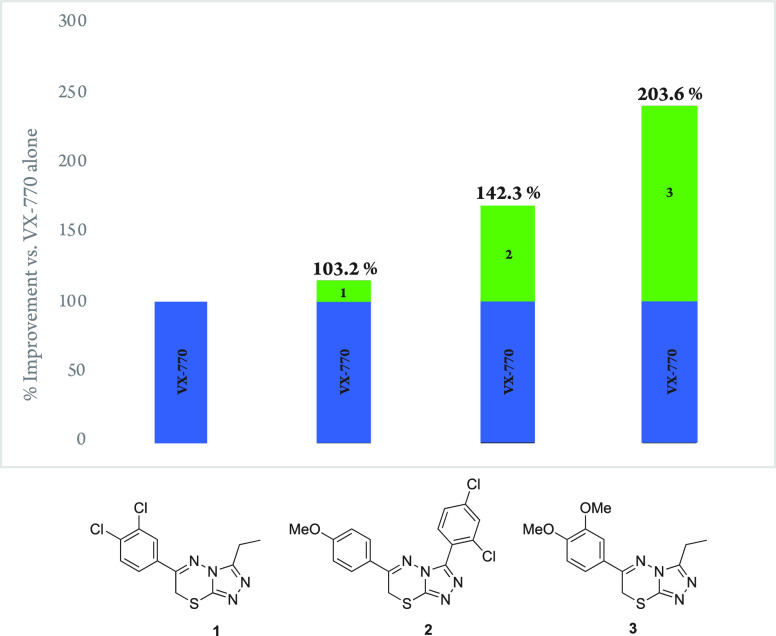
Commercially available compounds identified by high throughput screen. In conjunction with VX-770, these potentiate G551D CFTR up to 204% beyond the levels using VX-770 alone.

Among CFTR variants, G551D CFTR was selected for our SAR campaign for three reasons. First, G551D is typically viewed as a severe gating defect, and testing other compounds against this variant was viewed as a stringent means to assess bioactivity. Second, G551D was the prototypic mutation for which the first CFTR modulator, VX-770 (Figure 1), gained regulatory approval. Lastly, G551D is a relatively common defect and may benefit from combination treatment with drugs exhibiting a distinct mechanism of action, including those that augment CFTR function at saturating concentrations of VX-770. Importantly, a subset of patients encoding G551D show only marginal improvement following treatment with CFTR modulators, and new small molecules that augment G551D activity could be useful in this setting.

We performed a dose-dependence electrophysiology experiment on FRT cells expressing G551D CFTR using compound **3** (Supplemental Figure 3) in combination with VX-770 (1 μM) and found EC_50_ of ∼0.94 μM (95% CI of 0.70 μM to 1.24 μM) with maximal CFTR ion current approximately at 10 μM. Accordingly, analogs were tested, and their activities compared at 10 μM concentration in subsequent experiments. Throughout the study, analogue efficiency was expressed as percent gain above VX-770 alone. Our SAR evaluation focused on the synthesis of structural motifs common in the initial active hit series (Figure 2, **1**–**3**).

The bicyclic [1,2,4]triazolo[3,4-*b*]thiadiazine core of the confirmed, reordered hit compounds is a known pharmacophore with a wide array of published applications, from inhibition of fungal pyruvate kinase to antibiotic activity against *Mycobacterium tuberculosis*. Our synthetic approach to these analogs combined several approaches from the literature, resulting in a simple four-step synthesis starting from benzoic acid **4** (Figure 3).^22,23^ Condensation of **4** with hydrazine generated the hydrazide **5**, which on reaction with carbon disulfide and hydrazine generated the 1,2,4-triazole **6**. Condensation of **6** with an α-bromoketone generated [1,2,4]triazolo[3,4-*b*]thiadiazine (**7**–**10**). By modification of the benzoic acid and an α-bromoketone, a series of analogues could be prepared. We did not encounter unexpected or unusually high safety hazards.

**Figure 3:**
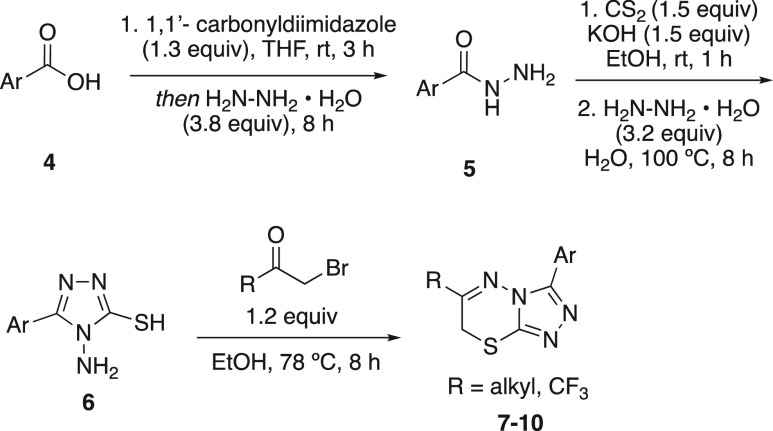
Synthetic approach to SAR campaign.

In our initial studies, we found that shifting the aryl ring to C3 and the ethyl group to C6 resulted in a drop in activity but demonstrated a viable starting point for conducting an SAR campaign. A series of 6-ethyl-3-aryl derivatives were evaluated to determine the optimum substituent on this ring (Figure 4). We found that a C3-appended dichloro- or dimethoxyarene ring led to a series of compounds that were efficacious in combination with VX-770 (Figure 4). Interestingly, the inclusion of a dimethoxy arene at C3 seemed to only slightly improve the activity of VX-770 (**7a**), despite the fact that this motif seemed to be important in the C6 aryl ring of hit-series compound **3**. An additional methoxy group on the ring (**7b**) led to a modest improvement in activity. In parallel with our investigation of methoxy-containing structures, we systematically explored the relationship between chlorine substitution on the ring and activity given that two of the confirmed hits (**1** and **3**) had appreciable activity but different substitution patterns. We were surprised to find that inclusion of only a single chlorine on the ring (**7c**) led to a similar improvement compared to **7b**. Moving the chlorine to a *meta*- rather than *para*-substitution demonstrated an increase in stimulation of the G551D current (**7d**). Appending an additional halide to the ring, as in compounds **7e**–**7h**, demonstrated that an increasing distance between the substituents, especially with an *ortho*-substituent, could lead to activity approaching or surpassing that of the original hit series.

**Figure 4:**
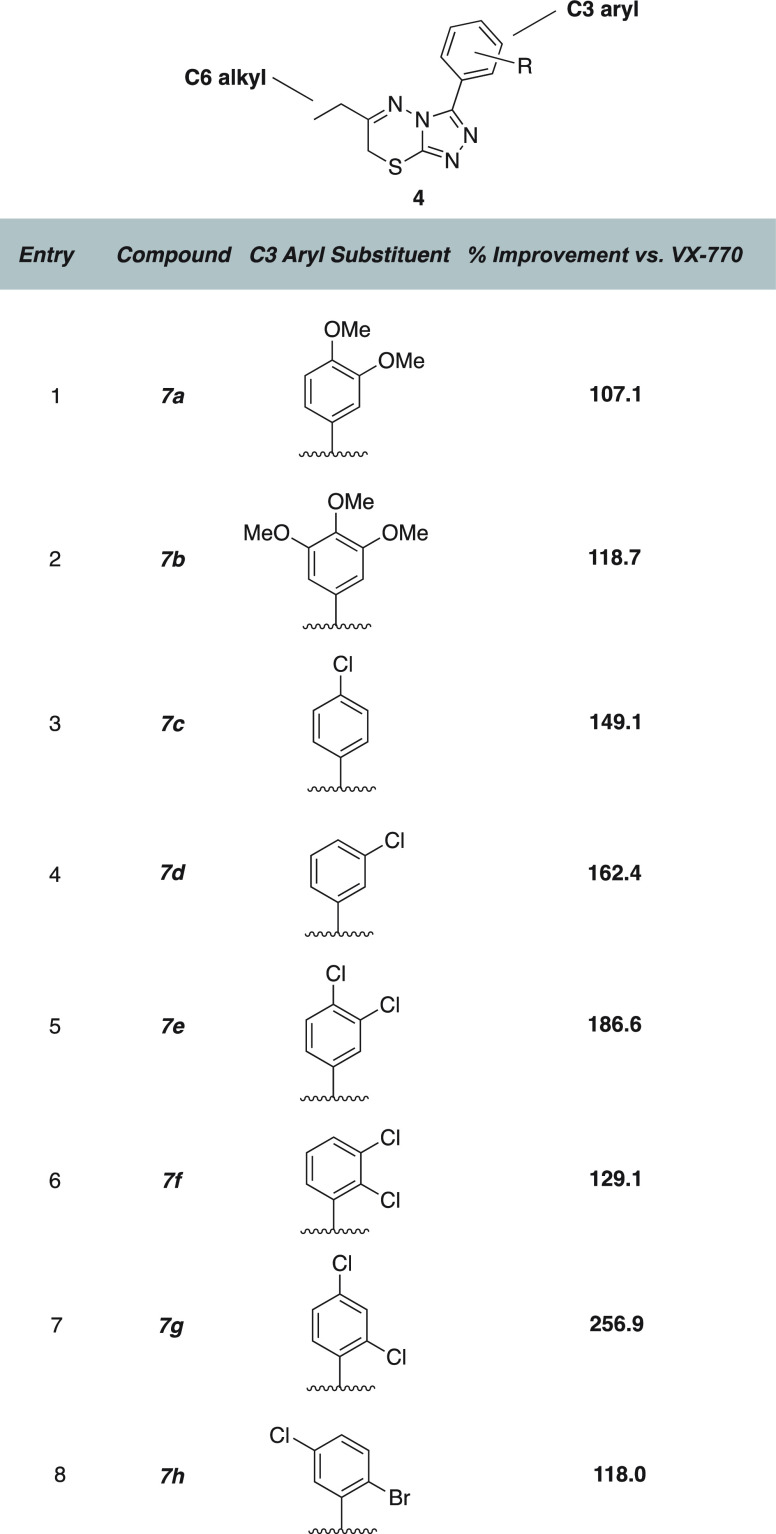
Initial SAR series, focused on the effects of C3 aryl substituents on co-potentiation of G551D CFTR.

We desired to probe the extent to which the identity of the C6 alkyl group contributed to activity (Figure 5), using two motifs, 2-bromo-5-chloro (**7h**) and 2,4-dichloro (**7g**), for initial studies. Holding the 2,4-dichloro arene constant, it seemed that increasing the size of the alkyl substituent corresponded to an increase in the compound’s co-potentiation of G551D CFTR, although an isopropyl group at C6 (**8c**) displayed a mild decrease in activity. The largest, *tert*-butyl (**8d**), far outpaced the original series, showing a close to 400% improvement in G551D stimulation when co-administered with the approved potentiator. This same general trend was present in the 2-bromo-5-chloro series with **9c** and **9d** showing the highest levels of activity.

**Figure 5:**
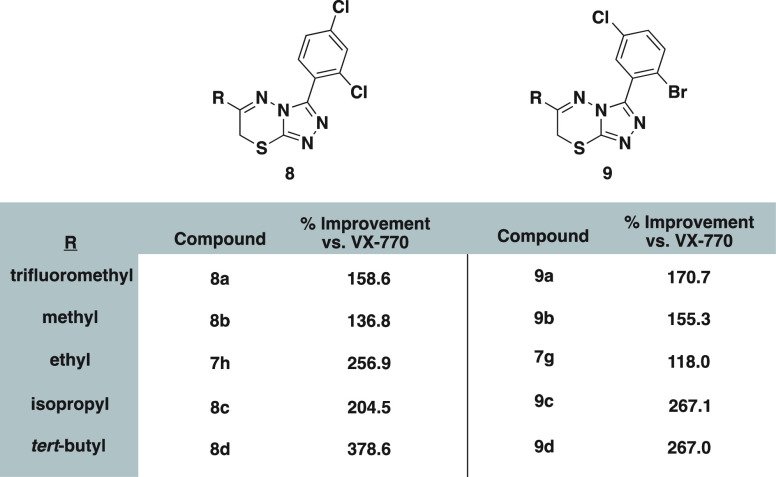
Investigation of the C6 alkyl moiety in relation to highly active C3 arene motifs.

While these original series in our own SAR campaign were promising, we were concerned about the ability of these compounds to be transitioned into further drug development, mainly due to the hydrophobicity of the most potent of the compounds, **8d** (clogP = 5.71) and **9d** (cLogP = 5.98). We investigated whether the inclusion of hydrogen-bond donors in the arene ring could improve the calculated water solubility of the compound series while maintaining or increasing efficacy in functional tests, although we were somewhat limited in this aspect by the highly nitrogenated triazolothiadiazine core. Compound **9c**, with an isopropyl at C6, was deemed a starting point since it demonstrated good activity along with lowered clogP (5.27) compared to **8d** (5.71) and **9d** (5.98).

When we replaced the 5-chloro substituent with a methoxy group, adding electron density into the ring as well as a hydrogen bond acceptor, activity slightly decreased to 232% G551D CFTR stimulation compared to VX-770 alone, although the cLogP dropped appreciably (**10a**) (Figure 6). Balancing this with a fluorine instead of an *ortho*-bromine maintained activity while lowering cLogP by a full unit (**10b**). A careful balance of electron density in the ring was clear: Adding an additional methoxy group at the 4-position (**10c**) degraded the activity to near that of VX-770 alone. Replacing the *ortho*-bromine with a methoxy group (**10d**) in conjunction with a 5-methoxy substituent showed a similar effect but was less detrimental to activity as seen in **10c**, negating the advantages of a lowered cLogP. This was consistent with the performance of other di- or trimethoxy arenes in our series (**7a**,**b**). Finally, we substituted the *ortho*-bromine for a chlorine. This compound, **10e**, showed a very favorable cLogP, with activity approaching 200% G551D CFTR stimulation. Generally, while it seemed that incorporation of elements that decreased cLogP could maintain the activity of compounds relative to the initial high-throughput screening hits, these same elements were not quite enough to stimulate the G551D CFTR to the same level as **8d** and **9d**.

**Figure 6:**
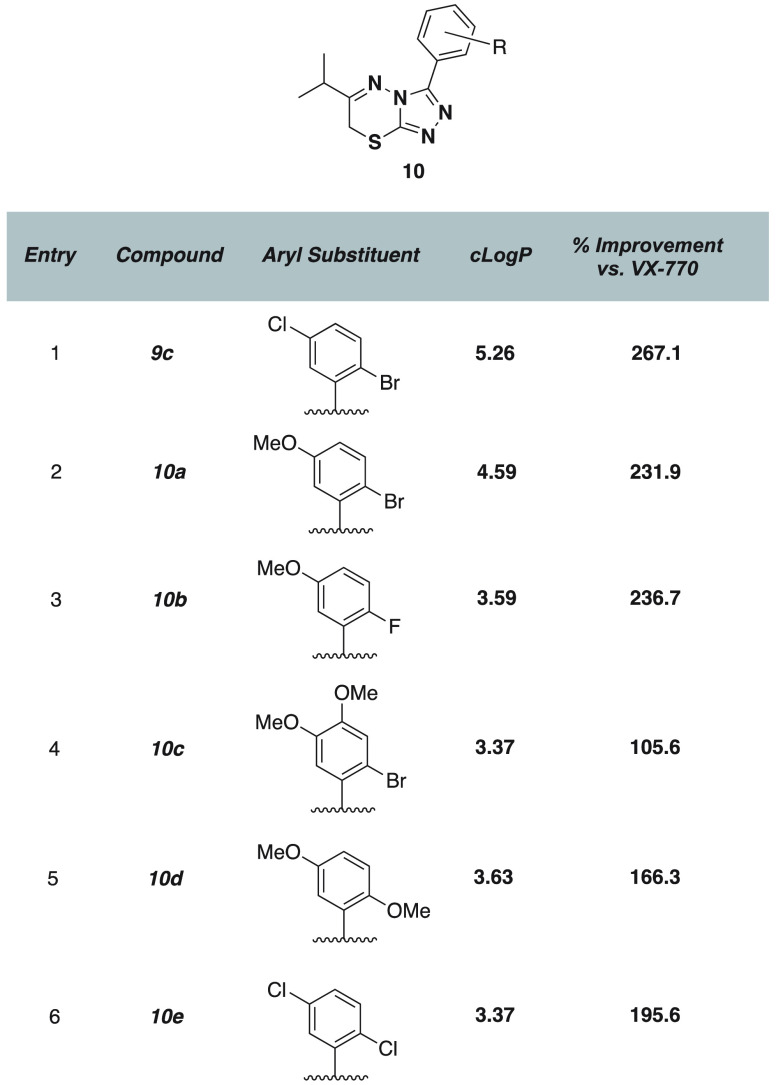
Optimization for solubility (cLogP) and potency together in co-potentiation of G551D CFTR.

In summary, the initial hit compound in our studies, **3**, was identified and confirmed by screening 300,000 compounds against mutant CFTR (N1303K) engineered to express an extracellularly directed enzymatic tag that allows surface protein detection by cell-based ELISA. We found from this screen that a primary characteristic of the compound class involves enhanced CFTR activation during co-administration with the approved CFTR potentiator (VX-770) (i.e., enhanced ion channel activity). Accordingly, we elected to optimize the strong effects on CFTR function in combination with VX-770 for the purpose of the present report.

An initial probing of the series produced by the high-throughput screen revealed that a small alkyl group at C6 of the triazolothiadiazine core and an arene at C3 produced compounds that co-potentiated mutant CFTR (G551D) to up to 257% more than with VX-770 alone (**7g**). Finding that a disubstituted arene, particularly with an *ortho*-substitution, was strongly correlated with efficacy, we varied the size of the C6 alkyl group. Interestingly, a slightly larger *tert*-butyl substituent produced compounds (**8d** and **9d**) that were quite potent, with up to 379% improvement compared to VX-770 alone. A reoptimization focused on balancing cLogP and efficacy in the biological assay produced 2 compounds (**10a**,**b**, **10e**) with cLogP < 5 and potency reaching and surpassing 200% CFTR function compared to VX-770 as a single agent. The ability of these novel compounds in our series to augment VX-770 stimulation of a relatively common CFTR variant (G551D) by close to 400% is surprising and indicates a distinct mechanism of action with potential usefulness against CFTR-related illnesses for patients who cannot obtain, tolerate, or adequately benefit from VX-770.

The recent advancement of CFTR modulators has provided remarkable clinical impact on care and overall prognosis among patients with CF.^24−26^ Currently approved CFTR potentiators and correctors are available in the United States for over 90% of the CF population.^27^ For the remaining ∼10%, many of whom encode rare or refractory CFTR variants, and for patients approved for modulator treatment who respond poorly, experience toxicity, or cannot afford the high cost of these drugs, alternative CFTR modulators represent a priority in the field. In this project, we describe a new class of small molecules that markedly increase the activity of mutant CFTRs treated with saturating concentrations of a prototypic and widely prescribed CFTR potentiator, VX-770. We characterized these compounds using the well- validated Fischer rat thyroid model that has been extensively utilized for cystic fibrosis drug discovery.^9,28−30^ All currently approved CFTR modulators, as well as several emerging compounds, have employed FRT polarized monolayers during drug development.^6,15^ For specific CFTR mutations and certain categories of pharmacologically active agents, FRT cells expressing mutant CFTRs have helped predict clinical benefit and contributed to regulatory approval. If results shown here can be confirmed in additional CF model systems (primary airway epithelial monolayers and CF-related tissue organoids), new agents based on this series could be tested as useful pharmacology adjuncts for CF and CFTR-related diseases such as chronic pancreatitis, rhinosinusitis, and chronic obstructive pulmonary disease.

## Supporting Information Available

Experimental procedures, NMR spectra, purity data, biological assay methods (PDF)

## Abbreviations

CFTR: cystic fibrosis transmembrane conductance regulator
CF: cystic fibrosis
HRP: horseradish peroxidase
FRT: Fischer rat thyroid
ELISA: enzyme-linked immunoassay
