# Supplementary material for: A Novel 7H-[1,2,4]Triazolo[3,4-b]thiadiazine-based Cystic Fibrosis Transmembrane Conductance Regulator Potentiator Directed toward Treatment of Cystic Fibrosis
Source: ACS Med Chem Lett. 2023 Sep 20;14(10):1338–43. doi: 10.1021/acsmedchemlett.3c00155 (PMC10577695; doi:10.1021/acsmedchemlett.3c00155)
Supplement: Supplementary file 1 — ml3c00155_si_001.pdf [file ml3c00155_si_001.pdf]

Supporting information for

A Novel 7H-[1,2,4]triazolo[3,4-b]thiadiazine-based CFTR Potentiator Directed Towards Treatment of Cystic Fibrosis

Andras Rab,[#a] Xun Yang,[#b] William F. Tracy,[b] Jeong S. Hong,[a] Disha Joshi,[a] Candela Manfredi,[a] Sadhana S. Ponnaluri,[a] Alexander A. Kolykhalov,[c] Min Qui,[d] Haian Fu,[d] Yuhong Du,*[d] Huw M. L. Davies,*[b] Eric J. Sorscher*[a]

[a] Department of Pediatrics, Emory University School of Medicine, Atlanta, Georgia 30322, United States

[b] Department of Chemistry, Emory University, 1515 Dickey Dr., Atlanta, Georgia 30329, United States

[c] Department Emory Institute for Drug Development, Atlanta, Georgia 30322, United States

[d] Department of Pharmacology and Chemical Biology, Emory University School of Medicine, Atlanta, GA 30322, USA; Emory Chemical Biology Discovery Center, Emory University School of Medicine, Atlanta, GA 30322, United States

[#] A.R. and X.Y. contributed equally to this study.

Contents

Supplemental Biological Data **S1**

Figure S1: Cell surface localized N1303K-HRP CFTR is increased by compound 3 in FRT cells S1

Figure S2: N1303K CFTR functional rescue by elexacaftor/tezacaftor/ivacaftor (ETI) is augmented by compound 3 in FRT cells S2

Figure S3: Dose-dependence and EC_{50} of compound 3 .... S2

Table S1: Functional characteristics of compounds on Fischer rat thyroid clonal cells stably expressing G551D CFTR. S3

Materials and Methods-Biological **S3**

Electrophysiology S4

Cell Culture S4

Materials and Methods-Chemical **S4**

Chemical Synthesis **S5**

Synthesis of Intermediates S5

Synthesis of Final Compounds S6

References **S12**

^{1}H NMR Spectra **S13**

^{13}C NMR Spectra **S23**

^{19}F NMR Spectra **S34**

Supplemental Biological Data

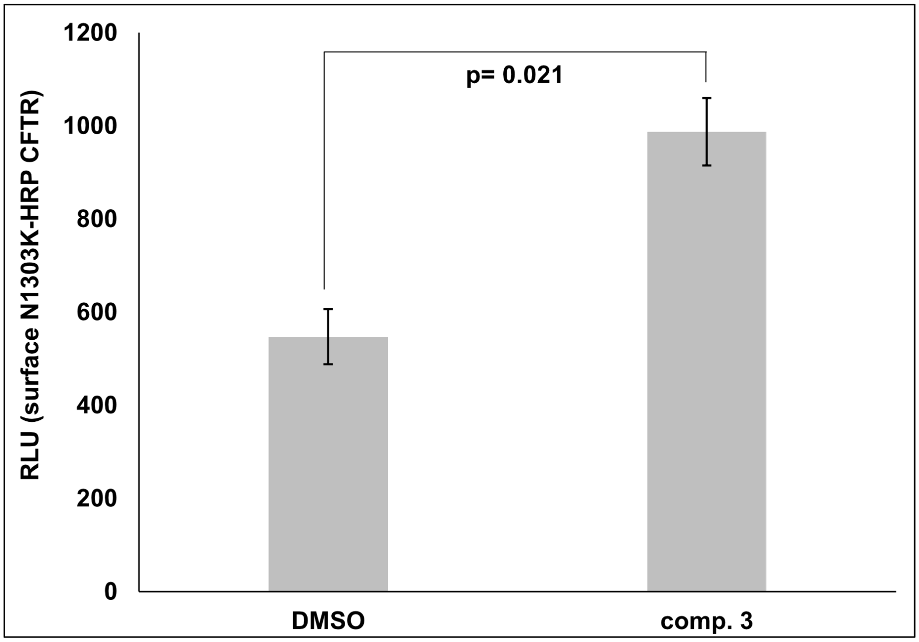

Supplemental Figure S1. Cell surface localized N1303K-HRP CFTR is increased by compound 3 in FRT cells. We observed elevated HRP signal indicating enhanced surface localization following compound 3 treatment (5µM; 24hrs) compared to vehicle control. Data is representative of 4 biological repeats and expressed as mean±SEM. Single-factor ANOVA was used to determine significance.

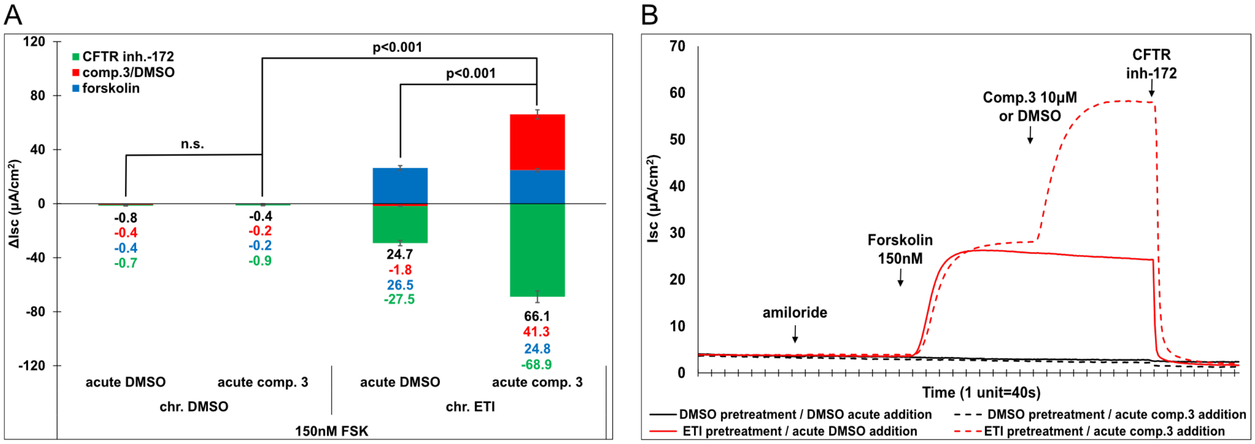

Supplemental Figure 2. N1303K CFTR functional rescue by elexacaftor/tezacaftor/ivacaftor (ETI) is augmented by compound 3 in FRT cells. Panel A. Subsequential addition of forskolin (FSK, 150nM) and compound 3 (10µM) significantly enhanced short circuit current (Isc) compared to vehicle control (DMSO) specifically in the setting of chronic ETI administration (24 hrs, 5µM elexacaftor, 3µM tezacaftor, 1µM ivacaftor). Bar graph depicts change in short circuit current (ΔIsc) (red, stimulated current by compound 3 or vehicle; blue, forskolin-stimulated current at saturating concentration; and green, inhibitable current by CFTR inh.-172). Data are presented as mean±SEM, n = 4-5 biological replicates per condition **Panel B** displays representative electrophysiology tracings (Ussing chamber). Wild type (wt) CFTR activation in this assay system is ~450 µA/cm^2. Isc above 10% wt activity is predictive of clinical benefit (Han, Rab et al., *JCI Insight*, 2018).

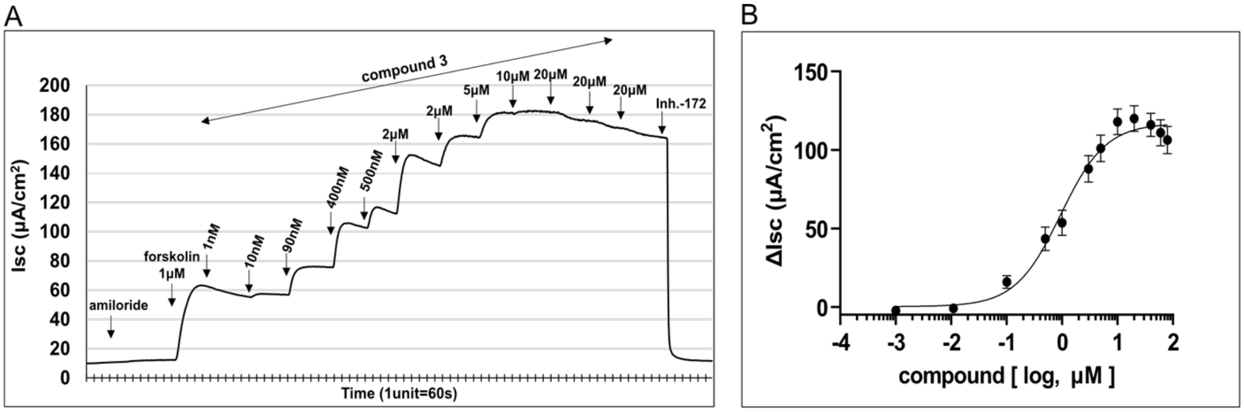

Supplemental Figure 3. Dose-dependence and EC$_{50}$ of compound 3. G551D CFTR is corrected by compound 3 in a dose-dependent manner when saturating concentrations of VX-770 are co-administered. (**Panel A**) EC$_{50}$ of the compound in combination with VX-770 = 940nM (95% CI of 696nM to 1244nM). Compound 3 as a single agent induces low level G551D activation (appx. 30µA/cm^2) at 10µM when cellular cAMP is minimal (5nM forskolin). (**Panel B**) GraphPad Prism 9 software was used for EC$_{50}$ calculation.

Supplemental Table 1: Functional characteristics of compounds on Fischer rat thyroid clonal cells stably expressing G551D CFTR.

compound ID	stimulated Isc (μA/cm^2)	% CFTR function compared to VX-770 alone	mean±SEM%	# of biological replicates (n)
**vehicle (DMSO)**	96.7	100.0	100±7.95%	22
**1**	99.8	103.2	103.2±7.35%	4
**2**	137.6	142.3	142.3±7.16%	3
**3**	196.9	203.6	203.6±7.23%	33
**7a**	103.6	107.1	107.1±14.62%	4
**7b**	114.8	118.7	118.7±9.39%	4
**7c**	144.5	149.4	149.4±8.28%	4
**7d**	157.1	162.4	162.4±5.98%	4
**7e**	180.5	186.6	186.6±6.56%	4
**7f**	125.0	129.2	129.2±9.81%	4
**7g**	248.5	256.9	256.9±10.34%	4
**7h**	114.1	118.0	118.0±6.34%	3
**8a**	153.6	158.8	158.8±13.43%	4
**8b**	136.8	141.5	141.5±18.26%	4
**8c**	197.8	204.5	204.5±5.89%	4
**8d**	366.2	378.6	378.6±7.61%	6
**9a**	165.1	170.7	170.7±5.63%	4
**9b**	150.2	155.3	155.3±8.68%	5
**9c**	258.3	267.1	267.1±10.10%	7
**9d**	258.2	267.0	267.0±11.27%	7
**10a**	189.2	195.6	195.6±6.30%	3
**10b**	224.3	231.9	231.9±6.18%	7
**10c**	160.9	166.3	166.3±5.13%	8
**10d**	102.2	105.6	105.6±6.03%	3
**10e**	228.9	236.7	236.7±4.20%	8

Materials and Methods-Biological

Cell-based cell surface HRP-CFTR ELISA assay

Fisher rat thyroid cells expressing N1303K-HRP CFTR were cultured on T-75 flasks. Cells were seeded onto Costar 96-well, white, clear flat bottom assay plates (Corning, 3903) 24 hours prior to compound treatment. The following day, fresh media containing 5μM test compound (positive drugs from high-throughput screen or vehicle control (DMSO)) was added. Twenty-four hours later, media was removed, and cells washed with PBS at room temperature. PBS was then replaced with 50μl SuperSignal ELISA Femto chemiluminescent substrate (ThermoFisher, 37074) in each well to determine cell surface HRP activity. Cell culture plates were assayed after a 2-minute incubation at room temperature using FlexStation 3 (Molecular Devices) equipment and relative luminescent signal determined. Each compound was studied using 4 biological replicates. Results were expressed as mean±SEM. Statistical significance was calculated with ANOVA for single-factor analysis.

Electrophysiology

In the course of the SAR study, monolayers were evaluated using an EasyMount Ussing Chamber System (Physiologic Instruments) and bathed apically in low chloride Ringer`s solution: 140mM Na-gluconate, 1.2mM NaCl, 25mM $NaHCO_3$, 3.33mM KH_2PO_4, 0.83mM K_2HPO_4, 1.2mM $CaCl_2$, 1.2mM $MgCl_2$, and 10mM D-glucose (pH 7.4). 120mM NaCl in the buffer at the basolateral surface was replaced with Na-gluconate to establish a chloride gradient. Temperature of bathing solutions was maintained at 37 °C and stirred by bubbling 5% CO_2/95% O_2. Investigated compounds were dissolved in DMSO at 10mM stock solutions and diluted/administered acutely during the assay, with modification as needed for specific protocols. Once base line stabilized, 100µM amiloride (MilliporeSigma, A7410) was applied to both apical and basolateral monolayer surfaces to inhibit epithelial sodium channel (ENaC) activity. CFTR protein was activated by applying 5µM forskolin (MilliporeSigma, F3917) to both sides of the monolayer followed by addition of 10µM test compound of interest. Maximal functional rescue by each compound was assessed in the presence of 5µM VX-770 (SelleckChem, S1144) apical addition. At the end of recording, CFTR (inh)-172 (10µM; MilliporeSigma C2992) was administered apically to inhibit CFTR-mediated current. Short circuit current (Isc) was measured under voltage clamp conditions and change in Isc calculated and expressed as mean ± standard error compared with vehicle control (DMSO).

Cell Culture

Fischer rat thyroid (FRT) cells expressing CFTR variants established by our laboratory (Han, Rab et al., *JCI Insight*, 2018) were cultured in Coon`s modification of Nutrient Mixture F-12 Ham (Sigma, F6636) supplemented with 2.68 g/l sodium-bicarbonate in the presence of 5% fetal bovine serum (Gibco). The cells were maintained at 37C° in a humidified atmosphere, with 5% CO_2 – 95% air. To establish polarized cell culture for functional studies, FRT cells were seeded onto Transwell permeable supports (Corning, 3470) and cultured for 5 days until a well polarized monolayer was formed (with transepithelial resistance of at least 400 $\Omega \times cm^2$).

Statistics

We applied single-factor ANOVA to determine significance levels between groups in experiments utilizing the HRP-based cell surface localization assay for N1303K CFTR. EC_{50} of compound 3 was determined with GraphPad Prism 9 software and the non-linear fit, sigmoidal, 4PL, X concentration function.

Materials and Methods-Chemical

All reagents were used as received from commercial suppliers, unless otherwise stated. Proton (^{1}H) NMR spectra were recorded at 400 MHz on a Varian-400 or Bruker-400, at 500 MHz on an Inova-500, or a 600 MHz Inova-600 spectrometer. Carbon-13 (^{13}C) NMR spectra were recorded at 150 MHz on an Inova-600 or Bruker-600 spectrometer, or 126 MHz on an Inova-500 spectrometer. Fluorine (^{19}F) NMR spectra were recorded at 282 MHz on an Inova-500 spectrometer. NMR spectra were recorded in deuterated chloroform ($CDCl_3$) or dimethylsulfoxide (DMSO-d^6) solutions, with residual chloroform (δ 7.26 ppm for ^{1}H NMR and δ 77.16 ppm for ^{13}C NMR) or residual dimethylsulfoxide (δ 2.50 for ^{1}H NMR and δ 39.52 ppm for ^{13}C NMR) as the internal standard and were reported in parts per million (ppm). Abbreviations for signal couplings are as follows: s, singlet; d, doublet; t, triplet; q, quartet; quin, quintet; sex, sextet; sep, septet;

and m, multiplet. Coupling constants were taken from the spectra directly and are uncorrected. Melting points (mp) were measured in open capillary tubes with a Mel-Temp Electrothermal melting points apparatus and are uncorrected. Mass spectrometric determinations were carried out on a Thermo Finnigan LTQ-FTMS spectrometer with nano-spray (NSI), electron-spray (ESI) or atmospheric pressure chemical ionization (APCI), using a Fourier transform ion cyclotron resonance (FT-ICR) mass analyzer. Analytical thin layer chromatography (TLC) was performed on silica gel plates using ultraviolet (UV) light, potassium permanganate stain, or cerium ammonium molybdate stain for visualization. Flash column chromatography was performed using Florisil® in hand-packed glass columns, or with silica gel 60 Å (230 – 400 mesh) on a Biotage Isolera Four MPLC-UV chromatograph using hand-packed silica gel columns, and Celite® as a dry-loading absorbent. Preparative high-performance liquid chromatography was performed on an Agilent Technologies 1260 Infinity Series chromatograph, using a mixed solution of HPLC-grade water (with 0.1% trifluoroacetic acid or TFA) and acetonitrile (with 0.1% TFA). Purity of final compounds was assessed by ^{1}H NMR. LogP values were calculated using ChemDraw 19®.

Caution! Upon addition of hydrazine hydrate after the reaction of carbon disulfide with the aryl hydrazide intermediate, the reaction evolves toxic H_2S gas. A reverse funnel trap with 2 M KOH solution must be employed to quench the evolved gas.

Chemical Synthesis

Route for the Synthesis of 7H-[1,2,4]triazolo[3,4-b]thiadiazines

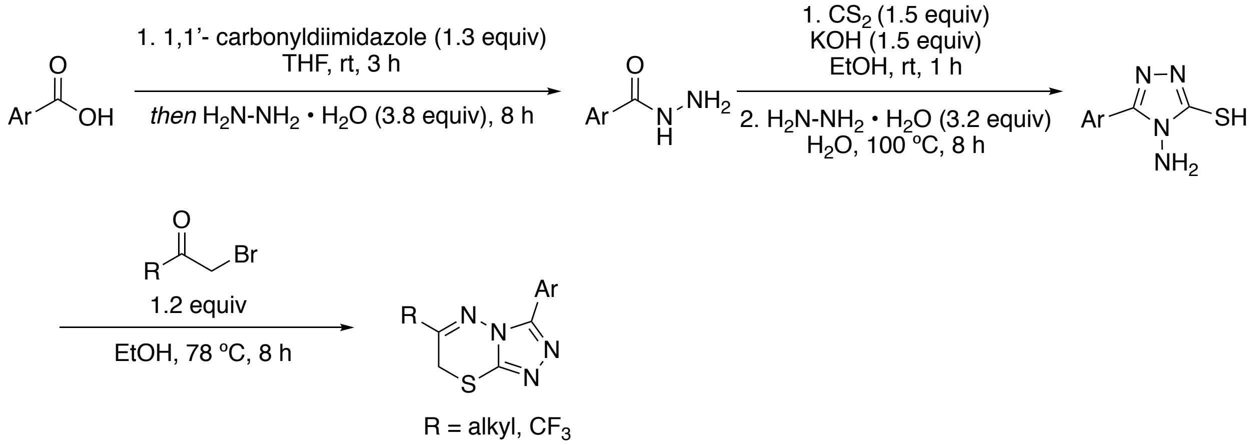

Synthesis of Intermediates

<u>General Procedure for the Preparation of Acid Hydrazides (GP 1)</u> Following an adapted literature procedure,[2] a round-bottom flask equipped with magnetic stir bar was flame dried under vacuum and backfilled with dry nitrogen. The carboxylic acid (1 Eq, 5.00 mmol) and 1,1-carbonyldiimidazole (CDI) (1.30 Eq, 6.50 mmol) were added and taken up in 15 mL dry tetrahydrofuran (THF). After stirring for 3h at room temperature, or until the cessation of apparent CO_2 evolution, hydrazine hydrate (2.33 mL, ~50% Wt, 3.83 Eq) in 5 mL THF was added dropwise via syringe. After stirring at room temperature overnight,

the reaction was concentrated in vacuuo. The crude residue was recrystallized from absolute ethanol to afford the acid hydrazide, which was used directly for the next reaction without further purification.

General Procedure for the Preparation of 4-amino-4H-1,2,3-triazole-3-thiols (GP 2) Following an adapted literature procedure,[2] potassium hydroxide (KOH) (1.5 Eq, 2.00 mmol) and the acid hydrazide (1.0 Eq, 1.50 mmol) were added to absolute ethanol (3.2 mL) and stirred until dissolution under ambient conditions. Neat carbon disulfide (1.50 Eq, 2.00 mmol) was added dropwise and the reaction was stirred vigorously for seven h. The precipitated solid was collected via vacuum filtration, washing with cold diethyl ether. After drying under high vacuum (<1 torr), the material was used in the next step without further purification. The solid along with water (270 µL) and hydrazine hydrate (470 µL, ~50% Wt, 3.20 Eq) were added to a round-bottom flask equipped with reflux condenser and reverse funnel trap and stirred vigorously overnight. The reaction was allowed to cool to room temperature, after which it was acidified to a pH of ~1 with 2N hydrochloric acid (HCl). The solid precipitate was collected by vacuum filtration, washing with water, affording the 4-amino-5-phenyl-4H-1,2,4-triazole-3-thiol, which was used directly for the next reaction without further purification.

Synthesis of Final Compounds

General Procedure for the Preparation of 1,2,4-Triazolo[3,4-b][1,3,4]Thiadiazine Derivatives (GP 3) According to a procedure adapted from the literature,[3] the 4-amino-5-substituted-4H-1,2,4-triazole-3-thiol (0.5 mmol, 1 equiv) and α-bromo-ketone (0.6 mmol, 1.1 equiv) were added to a round-bottom flask equipped with magnetic stir bar and reflux condenser. The reaction was refluxed in absolute ethanol (10 mL) for 8 h, after which it was concentrated in vacuuo and purified by flash column chromatography on Florisil® ($MeOH/CH_2Cl_2$ 0:100 to 5:95). Following this, the compound was further purified if necessary by preparative HPLC (10-70% acetonitrile/water with 0.1% 2,2,2-trifluoroacetic acid). Drying in a vacuum desiccator afforded the 1,2,4-triazolo[3,4-b][1,3,4]thiadiazine derivative.

3-(3,4-dimethoxyphenyl)-6-ethyl-7*H*-[1,2,4]triazolo[3,4-*b*][1,3,4]-thiadiazine (7a)

Following **GP3**, 4-amino-5-(3,4-dimethoxyphenyl)-4H-1, 2, 4-triazole-3-thiol (756 mg, 3.0 mmol), 1-bromo-2-butanone (498 mg, 3.3 mmol) were refluxed in 15 mL EtOH for 8 h. The reaction was then concentrated under vacuum and purified by flash column chromatography on Florisil® ($MeOH/CH_2Cl_2$ 0:100 to 5:95), affording **7a** (349 mg, 57% yield) as a white solid (mp 158 – 159 °C): ^1H NMR (600 MHz, DMSO-d_6) δ 7.63 – 7.59 (m, 2H), 7.12 (d, J = 8.9 Hz, 1H), 3.86 (s, 2H), 3.83 (s, 3H), 3.81 (s, 3H), 2.69 (q, J = 7.3 Hz, 2H), 1.20 (t, J = 7.3 Hz, 3H); ^{13}C NMR (151 MHz, DMSO-d_6) δ 162.7, 150.7, 150.3, 148.5, 141.5, 120.7, 118.5, 111.6, 110.7, 55.6, 55.5, 30.0, 24.3, 9.6; HRMS ($^+$APCI) (*m/z*): [M + H^+] calcd for $C_{14}H_{17}N_4O_2S$, 305.1067; found, 305.1065. Purity: >95% (^1H NMR).

3-(3,4,5-trimethoxyphenyl)-6-ethyl-7*H*-[1,2,4]triazolo[3,4-*b*][1,3,4]-thiadiazine (7b)

Following **GP3**, 4-amino-5-(3, 4, 5-trimethoxyphenyl)-4H-1, 2, 4-triazole-3-thiol (282 mg, 1.0 mmol), 1-bromo-2-butanone (166 mg, 1.1 mmol) were refluxed in 10 mL absolute ethanol for 8 h. The reaction was then concentrated under vacuum and purified by flash column chromatography on Florisil® ($MeOH/CH_2Cl_2$

0:100 to 5:95), affording **7b** (177 mg, 53% yield) as a white solid (mp 145 – 146 °C): ^{1}H NMR (600 MHz, DMSO-d_6) δ 7.38 (s, 2H), 3.87 (s, 2H), 3.84 (s, 6H), 3.74 (s, 3H), 2.71 (q, *J* = 7.2 Hz, 2H), 1.21 (t, *J* = 7.2 Hz, 3H); ^{13}C NMR (151 MHz, DMSO-d_6) δ 162.9, 152.8, 150.4, 142.0, 139.0, 121.4, 105.1, 60.2, 55.9, 30.1, 24.4, 9.5; HRMS ($^+$APCI) (*m/z*): [M + H$^+$] calcd for $C_{15}H_{19}N_4O_3S$, 335.1172; found, 335.1170. Purity: >95% (^{1}H NMR).

3-(4-chlorophenyl)-6-ethyl-7*H*-[1,2,4]triazolo[3,4-*b*][1,3,4]-thiadiazine (7c)

Following **GP3**, 4-amino-5-(4-chlorophenyl)-4H-1, 2, 4-triazole-3-thiol (113 mg, 0.5 mmol) and 1-bromo-2-butanone (83 mg, 0.55 mmol) were refluxed in 10 mL absolute ethanol for 8 h. The reaction was then concentrated under vacuum and purified by flash column chromatography on Florisil® (MeOH/CH_2Cl_2 0:100 to 5:95), affording **7c** (85 mg, 61% yield) as a white solid (mp 133 – 134 °C): ^{1}H NMR (600 MHz, DMSO-d_6) δ 8.04 – 8.01 (m, 2H), 7.65 – 7.62 (m, 2H), 3.89 (s, 2H), 2.68 (q, *J* = 7.3 Hz, 2H), 1.19 (t, *J* = 7.3 Hz, 3H); ^{13}C NMR (151 MHz, DMSO-d_6) δ 163.3, 150.0, 142.4, 134.9, 129.3, 128.8, 124.9, 30.0, 24.2, 9.7; HRMS ($^+$APCI) (*m/z*): [M + H$^+$] calcd for $C_{12}H_{12}ClN_4S$, 279.0466; found, 279.0466. Purity: >95% (^{1}H NMR).

3-(3-chlorophenyl)-6-ethyl-7*H*-[1,2,4]triazolo[3,4-*b*][1,3,4]-thiadiazine (7d)

Following **GP3**, 4-amino-5-(3-chlorophenyl)-4H-1, 2, 4-triazole-3-thiol (113 mg, 0.5 mmol) and 1-bromo-2-butanone (83 mg, 0.55 mmol) were refluxed in 10 mL absolute ethanol for 8 h. The reaction was then concentrated under vacuum and purified by flash column chromatography on Florisil® (MeOH/CH_2Cl_2 0:100 to 5:95), affording **7d** (73 mg, 53% yield) as a white solid (mp 98 – 99 °C): ^{1}H NMR (600 MHz, DMSO-d_6) δ 8.06 – 8.05 (m, 1H), 7.99 (dt, *J* = 6.7, 1.8 Hz, 1H), 7.62 – 7.58 (m, 2H), 3.89 (s, 2H), 2.70 (q, *J* = 7.2 Hz, 2H), 1.20 (t, *J* = 7.3 Hz, 3H; ^{13}C NMR (151 MHz, DMSO-d_6) δ 163.4, 149.6, 142.6, 133.3, 130.7, 129.9, 127.9, 127.1, 126.1, 30.0, 24.3, 9.5; HRMS ($^+$APCI) (*m/z*): [M + H$^+$] calcd for $C_{12}H_{12}ClN_4S$, 279.0466; found, 279.0463. Purity: >95% (^{1}H NMR).

3-(3,4-dichlorophenyl)-6-ethyl-7*H*-[1,2,4]triazolo[3,4-*b*][1,3,4]-thiadiazine (7e)

Following **GP3**, 4-amino-5-(3, 4-dichlorophenyl)-4H-1, 2, 4-triazole-3-thiol (780 mg, 3.0 mmol) and 1-bromo-2-butanone (498 mg, 3.3 mmol) were refluxed in 15 mL absolute ethanol for 8 h. The reaction was then concentrated under vacuum and purified by flash column chromatography on Florisil® (MeOH/CH_2Cl_2 0:100 to 5:95), affording **7e** (812 mg, 87% yield) as a white solid (mp 205 – 206 °C): ^{1}H NMR (600 MHz, DMSO-d_6) δ 8.26 (d, *J* = 2.0 Hz, 1H), 8.02 (dd, *J* = 8.5, 2.0 Hz, 1H), 7.85 (d, *J* = 8.5 Hz, 1H), 3.89 (s, 2H), 2.71 (q, *J* = 7.2 Hz, 2H), 1.21 (t, *J* = 7.2 Hz, 3H); ^{13}C NMR (151 MHz, DMSO-d_6) δ 164.1, 149.3, 143.3, 133.3, 132.0, 131.6, 129.5, 128.0, 126.9, 30.5, 24.8, 10.0; HRMS ($^+$APCI) (*m/z*): [M + H$^+$] calcd for $C_{12}H_{11}Cl_2N_4S$, 313.0067; found 313.0081. Purity: >95% (^{1}H NMR).

3-(2,3-dichlorophenyl)-6-ethyl-7*H*-[1,2,4]triazolo[3,4-*b*][1,3,4]-thiadiazine (7f)

Following **GP3**, 4-amino-5-(2, 3-dichlorophenyl)-4H-1, 2, 4-triazole-3-thiol (130 mg, 0.5 mmol) and 1-bromo-2-butanone (91 mg, 0.6 mmol) were refluxed in 10 mL absolute ethanol for 8 h. The reaction was

then concentrated under vacuum and purified by flash column chromatography on Florisil® (MeOH/CH_2Cl_2 0:100 to 5:95), affording **7f** (121 mg, 87% yield) as a white solid (mp 95 – 96 °C): ^{1}H NMR (600 MHz, DMSO-*d*$_6$) δ 7.87 (dd, *J* = 8.0, 1.6 Hz, 1H), 7.59 (dd, *J* = 7.7, 1.6 Hz, 1H), 7.54 (t, *J* = 7.9 Hz, 1H), 3.92 (s, 2H), 2.56 (q, *J* = 7.3 Hz, 2H), 1.06 (t, *J* = 7.3 Hz, 3H); ^{13}C NMR (151 MHz, DMSO-*d*$_6$) δ 163.1, 149.7, 141.8, 132.6, 132.4, 131.5, 131.2, 128.4, 127.9, 29.9, 24.5, 9.8; HRMS ($^+$APCI) (*m/z*): [M + H$^+$] calcd for $C_{12}H_{11}Cl_2N_4S$, 313.0076; found, 313.0072. Purity: >95% (^{1}H NMR).

3-(2-bromo-5-chlorophenyl)-6-ethyl-7*H*-[1,2,4]triazolo[3,4-*b*][1,3,4]-thiadiazine (7g)

Following **GP3**, 4-amino-5-(2-bromo-5-chlorophenyl)-4H-1, 2, 4-triazole-3-thiol (152 mg, 0.5 mmol) and 1-bromo-2-butanone (91 mg, 0.6 mmol) were refluxed in 10 mL absolute ethanol for 8 h. The reaction was then concentrated under vacuum and purified by flash column chromatography on Florisil® (MeOH/CH_2Cl_2 0:100 to 5:95), affording **7g** (97 mg, 54% yield) as a white solid (mp 188 – 189 °C): ^{1}H NMR (600 MHz, DMSO-*d*$_6$) δ 7.84 (d, *J* = 8.6 Hz, 1H), 7.69 (d, *J* = 2.6 Hz, 1H), 7.59 (dd, *J* = 8.6, 2.6 Hz, 1H), 3.88 (s, 2H), 2.57 (q, *J* = 7.3 Hz, 2H), 1.07 (t, *J* = 7.3 Hz, 3H); ^{13}C NMR (151 MHz, DMSO-*d*$_6$) δ 163.3, 150.1, 141.8, 134.7, 132.4, 132.2, 132.1, 129.4, 121.6, 29.9, 24.7, 9.8; HRMS ($^+$APCI) (*m/z*): [M + H$^+$] calcd for $C_{12}H_{11}BrClN_4S$, 356.9571; found 356.9574. Purity: >95% (^{1}H NMR).

3-(2,4-dichlorophenyl)-6-ethyl-7*H*-[1,2,4]triazolo[3,4-*b*][1,3,4]-thiadiazine (7h)

Following **GP3**, 4-amino-5-(2, 4-dichlorophenyl)-4H-1, 2, 4-triazole-3-thiol (130 mg, 0.5 mmol), 1-bromo-2-butanone (91 mg, 0.6 mmol) were refluxed in 10 mL absolute ethanol for 8 h. The reaction was then concentrated under vacuum and purified by flash column chromatography on Florisil® (MeOH/CH_2Cl_2 0:100 to 5:95), affording **7h** (92 mg, 59% yield) as a white solid (mp 142 – 143 °C): ^{1}H NMR (600 MHz, DMSO-*d*$_6$) δ 7.86 (d, *J* = 1.7 Hz, 1H), 7.65 – 7.61 (m, 2H), 3.90 (s, 2H), 2.56 (q, *J* = 7.3 Hz, 2H), 1.07 (t, *J* = 7.3 Hz, 3H); ^{13}C NMR (151 MHz, DMSO-*d*$_6$) δ 163.0, 149.2, 141.8, 136.1, 134.3, 133.7, 129.5, 127.6, 124.5, 29.9, 24.5, 9.8; HRMS ($^+$APCI) (*m/z*): [M + H$^+$] calcd for $C_{12}H_{11}Cl_2N_4S$, 313.0076; found 313.0072. Purity: >95% (^{1}H NMR).

3-(2,4-dichlorophenyl)-6-trifluoromethyl-7*H*-[1,2,4]triazolo[3,4-*b*][1,3,4]-thiadiazine (8a)

Following **GP3**, 4-amino-5-(2, 4-dichlorophenyl)-4H-1, 2, 4-triazole-3-thiol (520 mg, 2.0 mmol) and 3-bromo-1,1,1-trifluoroacetone (420 mg, 2.2 mmol) were refluxed in absolute ethanol for 8 h. The reaction was then cooled to room temperature, then stored at a -20 °C freezer overnight. The precipitated white solid was collected by vacuum filtration, and the filter cake was washed with 30 mL cold Et_2O and 30 mL cold hexanes. The material was dried, affording **8a** (439 mg, 62% yield) as a white solid (mp 156 – 157 °C): ^{1}H NMR (600 MHz, DMSO-*d*$_6$) δ 8.13 – 7.82 (m, 1H), 7.81 – 7.51 (m, 2H), 4.39 (s, 2H); ^{13}C NMR (151 MHz, DMSO-*d*$_6$) δ 149.9, 145.5 (q, *J* = 36.1 Hz), 141.9, 136.8, 134.2, 133.9, 129.8, 127.8, 123.2, 119.2 (q, *J* = 275.9 Hz), 20.6; ^{19}F NMR (282 MHz, DMSO-*d*$_6$) δ -74.24. HRMS ($^+$APCI) (*m/z*): [M + H$^+$] calcd for $C_{11}H_6Cl_2F_3N_4S$, 352.9637; found 352.9635. Purity: >95% (^{1}H NMR).

3-(2,4-dichlorophenyl)-6-methyl-7*H*-[1,2,4]triazolo[3,4-*b*][1,3,4]-thiadiazine (8b)

Following **GP3**, 4-amino-5-(2, 4-dichlorophenyl)-4H-1, 2, 4-triazole-3-thiol (130 mg, 0.5 mmol) and chloroacetone (56 mg, 0.6 mmol) were refluxed in 10 mL absolute ethanol for 8 h. The reaction was then concentrated under vacuum and purified by flash column chromatography on Florisil® (MeOH/CH_2Cl_2 0:100 to 5:95), affording **8b** (140 mg, 94% yield) as a white solid (mp 196 – 197 °C): ^{1}H NMR (600 MHz, DMSO-d_6) δ 7.86 (dd, J = 1.4, 0.9 Hz, 1H), 7.64 – 7.60 (m, 2H), 3.89 (s, 2H), 2.23 (s, 3H); ^{13}C NMR (151 MHz, DMSO-d_6) δ 159.5, 149.1, 141.4, 136.1, 134.4, 133.7, 129.5, 127.6, 124.6, 25.4, 23.2; HRMS ($^+$APCI) (*m/z*): [M + H$^+$] calcd for $C_{11}H_9Cl_2N_4S$, 298.9919; found, 298.9917. Purity: >95% (^{1}H NMR).

3-(2,4-dichlorophenyl)-6-isopropyl-7*H*-[1,2,4]triazolo[3,4-*b*][1,3,4]-thiadiazine (8c)

Following **GP3**, 4-amino-5-(2, 4-dichlorophenyl)-4H-1, 2, 4-triazole-3-thiol (130 mg, 0.5 mmol) and 1-bromo-3-methylbutan-2-one (98 mg, 0.6 mmol) were refluxed in 10 mL absolute ethanol for 8 h. The reaction was then concentrated under vacuum and purified by flash column chromatography on Florisil® (MeOH/CH_2Cl_2 0:100 to 5:95), affording **8c** (162 mg, 99% yield) as a white solid (mp 87 – 88 °C): ^{1}H NMR (600 MHz, DMSO-d_6) δ 7.86 (d, J = 2.0 Hz, 1H), 7.66 (d, J = 8.3 Hz, 3H), 7.63 (dd, J = 8.3, 2.0 Hz, 1H), 3.94 (s, 2H), 2.80 (p, J = 6.8 Hz, 1H), 1.13 (d, J = 6.8 Hz, 6H); ^{13}C NMR (151 MHz, DMSO-d_6) δ 166.0, 149.4, 142.3, 136.1, 134.2, 133.6, 129.5, 127.6, 124.5, 35.5, 23.2, 18.9; HRMS ($^+$APCI) (*m/z*): [M + H$^+$] calcd for $C_{13}H_{13}Cl_2N_4S$, 327.0232; found, 327.0231. Purity: >95% (^{1}H NMR).

3-(2,4-dichlorophenyl)-6-*tert*-butyl-7*H*-[1,2,4]triazolo[3,4-*b*][1,3,4]-thiadiazine (8d)

Following **GP3**, 4-amino-5-(2, 4-dichlorophenyl)-4H-1, 2, 4-triazole-3-thiol (130 mg, 0.5 mmol) and 1-chloro-3, 3-dimethyl-butan-2-one (81 mg, 0.6 mmol) were refluxed in 10 mL absolute ethanol for 8 h. The reaction was then concentrated under vacuum and purified by flash column chromatography on Florisil® (MeOH/CH_2Cl_2 0:100 to 5:95), affording **8d** (155 mg, 91% yield) as a white solid (mp 132 – 133 °C): ^{1}H NMR (600 MHz, DMSO-d_6) δ 7.86 (d, J = 2.0 Hz, 1H), 7.67 (d, J = 8.3 Hz, 1H), 7.63 (dd, J = 8.3, 2.1 Hz, 1H), 4.00 (s, 2H), 1.17 (s, 9H); ^{13}C NMR (151 MHz, DMSO-d_6) δ 167.7, 149.6, 142.6, 136.0, 134.1, 133.6, 129.6, 127.5, 124.4, 39.0, 26.5, 21.9; HRMS ($^+$APCI) (*m/z*): [M + H$^+$] calcd for $C_{14}H_{15}Cl_2N_4S$, 341.0389; found 341.0388. Purity: >95% (^{1}H NMR).

3-(2-bromo-5-chlorophenyl)-6-trifluoromethyl-7*H*-[1,2,4]triazolo[3,4-*b*][1,3,4]-thiadiazine (9a)

Following **GP3**, 4-amino-5-(2-bromo-5-chlorophenyl)-4H-1, 2, 4-triazole-3-thiol (152 mg, 0.5 mmol), 3-bromo-1,1,1-trifluoroacetone (115 mg, 0.6 mmol) were refluxed in 10 mL absolute ethanol for 8 h. The reaction was then concentrated under vacuum and purified by flash column chromatography on Florisil® (MeOH/CH_2Cl_2 0:100 to 5:95), affording **9a** (179 mg, 90% yield) as a white solid (mp 156 – 157 °C): ^{1}H NMR (600 MHz, DMSO-d_6) δ 7.88 (d, J = 8.6 Hz, 1H), 7.73 (d, J = 2.6 Hz, 1H), 7.64 (dd, J = 8.6, 2.6 Hz, 1H), 4.37 (s, 2H); ^{13}C NMR (151 MHz, DMSO-d_6) δ 150.7 , 145.7 (q, J = 36.1 Hz), 141.8 , 134.9, 132.6, 132.5, 132.4, 128.0, 121.3, 119.1 (q, J = 275.9 Hz), 20.7; ^{19}F NMR (282 MHz, DMSO-d_6) δ -74.26. HRMS ($^+$APCI) (*m/z*): [M + H$^+$] calcd for $C_{11}H_6BrClF_3N_4S$, 396.9132; found, 396.9144. Purity: >95% (^{1}H NMR).

3-(2-bromo-5-chlorophenyl)-6-methyl-7*H*-[1,2,4]triazolo[3,4-*b*][1,3,4]-thiadiazine (9b)

Following **GP3**, 4-amino-5-(2-bromo-5-chlorophenyl)-4H-1, 2, 4-triazole-3-thiol (152 mg, 0.5 mmol) and chloroacetone (56 mg, 0.6 mmol) were refluxed in 10 mL absolute ethanol for 8 h. The reaction was then concentrated under vacuum and purified by flash column chromatography on Florisil® (MeOH/CH_2Cl_2 0:100 to 5:95), affording **9b** (155 mg, 91% yield) as a white solid (mp 206 – 207 °C): ^{1}H NMR (600 MHz, DMSO-d_6) δ 7.84 (d, J = 8.6 Hz, 1H), 7.68 (d, J = 2.6 Hz, 1H), 7.60 (dd, J = 8.6, 2.6 Hz, 1H), 3.87 (s, 2H), 2.24 (s, 3H); ^{13}C NMR (151 MHz, DMSO-d_6) δ 159.8, 150.0, 141.4, 134.7, 132.4, 132.2, 132.2, 129.6, 121.8, 25.5, 23.4; HRMS ($^+$APCI) (*m/z*): [M + H$^+$] calcd for $C_{11}H_9BrClN_4S$, 342.9414; found, 342.9418. Purity: >95% (^{1}H NMR).

3-(2-bromo-5-chlorophenyl)-6-isopropyl-7*H*-[1,2,4]triazolo[3,4-*b*][1,3,4]-thiadiazine (9c)

Following **GP3**, 4-amino-5-(2-bromo-5-chlorophenyl)-4H-1, 2, 4-triazole-3-thiol (152 mg, 0.5 mmol) and 1-bromo-3-methylbutan-2-one (98 mg, 0.6 mmol) were refluxed in 10 mL absolute ethanol for 8 h. The reaction was then concentrated under vacuum and purified by flash column chromatography on Florisil® (MeOH/CH_2Cl_2 0:100 to 5:95), affording 160 mg (86%) **9c** as a white solid (mp 122 – 123 °C): IR (film) 2970, 2931, 2874 cm^{-1}; ^{1}H NMR (600 MHz, DMSO-d_6) δ 7.85 (d, J = 8.6 Hz, 1H), 7.72 (d, J = 2.6 Hz, 1H), 7.61 (dd, J = 8.6, 2.7 Hz, 1H), 3.93 (s, 2H), 2.81 (hept, J = 6.7 Hz, 1H), 1.13 (d, J = 6.8 Hz, 6H); ^{13}C NMR (151 MHz, DMSO-d_6) δ 166.4, 150.2, 142.3, 134.8, 132.4, 132.2, 132.1, 129.3, 121.5, 35.5, 23.4, 19.0 ; HRMS (+APCI) (*m/z*): [M + H+] calcd for $C_{13}H_{13}BrClN_4S$ 370.9727, found 370.9731. Purity: >95% (^{1}H NMR).

3-(2-bromo-5-chlorophenyl)-6-*tert*-butyl-7*H*-[1,2,4]triazolo[3,4-*b*][1,3,4]-thiadiazine (9d)

Following **GP3**, 4-amino-5-(2-bromo-5-chlorophenyl)-4H-1, 2, 4-triazole-3-thiol (152 mg, 0.5 mmol) and 1-chloro-3,3-dimethyl-butan-2-one (81 mg, 0.6 mmol) were refluxed in 10 mL absolute ethanol for 8 h. The reaction was then concentrated under vacuum and purified by flash column chromatography on Florisil® (MeOH/CH_2Cl_2 0:100 to 5:95), affording **9d** (178 mg, 93% yield) as a white solid (mp 155 – 156 °C): ^{1}H NMR (600 MHz, DMSO-d_6) δ 7.84 (d, J = 8.6 Hz, 1H), 7.72 (d, J = 2.6 Hz, 1H), 7.59 (dd, J = 8.6, 2.6 Hz, 1H), 3.98 (s, 2H), 1.17 (s, 9H); ^{13}C NMR (151 MHz, DMSO-d_6) δ 167.9, 150.2, 142.5, 134.7, 132.3, 132.1, 131.9, 129.1, 121.3, 26.5, 22.1; HRMS ($^+$APCI) (*m/z*): [M + H$^+$] calcd for $C_{14}H_{15}BrClN_4S$, 384.9884; found, 384.9890. Purity: >95% (^{1}H NMR).

3-(2-bromo-5-methoxyphenyl)-6-isopropyl-7*H*-[1,2,4]triazolo[3,4-*b*][1,3,4]-thiadiazine (10a)

Following **GP3**, 4-amino-5-(2-bromo-5-methoxyphenyl)-4H-1, 2, 4-triazole-3-thiol (150 mg, 0.5 mmol) and 1-bromo-3-methylbutan-2-one (98 mg, 0.6 mmol) were refluxed in 10 mL absolute ethanol for 8 h. The reaction was then concentrated under vacuum and purified by flash column chromatography on Florisil® (MeOH/CH_2Cl_2 0:100 to 5:95), affording **10a** (151 mg, 82% yield) as a white solid (mp 111 – 112 °C): ^{1}H NMR (600 MHz, DMSO-*d6*) δ 7.67 (d, J = 8.9 Hz, 1H), 7.16 (d, J = 3.1 Hz, 1H), 7.09 (dd, J = 8.9, 3.1 Hz, 1H), 3.91 (s, 2H), 3.80 (s, 3H), 2.80 (h, J = 6.8 Hz, 1H), 1.13 (d, J = 6.8 Hz, 6H); ^{13}C NMR (151 MHz, DMSO-*d6*) δ 165.9, 158.3, 151.2, 141.9, 133.8, 128.4, 118.2, 117.8, 113.1, 55.7, 35.5, 23.3, 18.9. HRMS ($^+$APCI) (*m/z*): [M + H$^+$] calcd for $C_{14}H_{16}BrN_4OS$, 367.0223; found, 367.0220. Purity: >95% (^{1}H NMR).

3-(2-fluoro-5-methoxyphenyl)-6-isopropyl-7H-[1,2,4]triazolo[3,4-b][1,3,4]thiadiazine (10b)

Following **GP3**, 4-amino-5-(2-bromo-5-methoxyphenyl)-4H-1, 2, 4-triazole-3-thiol (500 mg, 2.08 mmol) and 1-bromo-3-methylbutan-2-one (412 mg, 2.50 mmol) were refluxed in 41 mL absolute ethanol for 8 h. The reaction was then concentrated under vacuum and purified by preparative HPLC (10-40% acetonitrile/water, 0.1% TFA), affording **10b** (335 mg, 42% yield) as a white semisolid: ^{1}H NMR (400 MHz, $CDCl_3$) δ 7.19 (dd, *J* = 5.3, 3.2 Hz, 1H), 7.03 (t, *J* = 9.2 Hz, 1H), 6.94 (ddd, *J* = 9.1, 4.0, 3.2 Hz, 1H), 3.75 (s, 3H), 3.49 (s, 2H), 2.78 (hept, *J* = 6.8 Hz, 1H), 1.17 (d, *J* = 6.9 Hz, 6H); ^{13}C NMR (101 MHz, $CDCl_3$) δ 163.5, 155.5, 155.4, 154.3 (d, *J* = 246 Hz), 149.5 (d, *J* = 2.3 Hz), 142.7, 118.4 (d, *J* = 8.0 Hz), 116.8 (d, *J* = 23.5 Hz), 114.7 (d, *J* = 2.3 Hz), 114.5 (d, *J* = 15.4 Hz), 55.8, 36.1, 29.6, 23.7, 19.3; ^{19}F NMR: -124.04 (*J* = 9.43, 4.78). HRMS ($^+$APCI) (*m/z*): [M + H$^+$] calcd for $C_{14}H_{16}ON_4F^{32}S$, 307.1023; found, 307.1017. Purity: >95% (^{1}H NMR).

3-(2-bromo-4,5-dimethoxyphenyl)-6-isopropyl-7H-[1,2,4]triazolo[3,4-b][1,3,4]thiadiazine (10c)

Following **GP3**, 4-amino-5-(2-bromo-4,5-dimethoxyphenyl)-4H-1, 2, 4-triazole-3-thiol (165 mg, 0.5 mmol) and 1-bromo-3-methylbutan-2-one (98 mg, 0.6 mmol) were refluxed in 10 mL absolute ethanol for 8 h. The reaction was then concentrated under vacuum and purified by flash column chromatography on Florisil® (MeOH/CH_2Cl_2 0:100 to 5:95), affording **10c** (40 mg, 20% yield) as a white solid (mp = 99-102 ºC): ^{1}H NMR (400 MHz, Chloroform-*d*) δ 7.13 (s, 1H), 7.09 (s, 1H), 3.92 (s, 3H), 3.92 (s, 3H), 3.46 (s, 2H), 2.84 (hept, *J* = 7.0 Hz, 1H), 1.23 (d, *J* = 6.9 Hz, 6H). ^{13}C NMR (101 MHz, $CDCl_3$) δ 163.8, 152.8, 151.1, 148.3, 142.2, 119.7, 115.8, 114.7, 56.3, 36.3, 29.8, 23.9, 19.4. HRMS ($^+$APCI) (*m/z*): [M + H$^+$] calcd for $C_{15}H_{18}O_2N_4{}^{79}Br^{32}S$, 397.0328; found, 397.0339. Purity: >95% (^{1}H NMR)

3-(2,5-dimethoxyphenyl)-6-isopropyl-7H-[1,2,4]triazolo[3,4-b][1,3,4]thiadiazine (10d) Following **GP 3** 4-amino-5-(2,5-dimethoxyphenyl)-4H-1,2,4-triazole-3-thiol (500 mg, 1.98 mmol) and 1-bromo-3-methylbutan-2-one (392 mg, 2.38 mmol) were refluxed in ethanol (39 mL) overnight. The compound was purified by flash column chromatography (10% MeOH in CH_2Cl_2, SiO_2), followed by preparative HPLC (10-40% acetonitrile/water, 0.1% TFA), giving **10d** (518 mg, 82% yield) as a white semisolid: ^{1}H NMR (600 MHz, Chloroform-*d*) δ 7.18 – 7.17 (d, *J* = 3.0 Hz, 1H), 7.04 – 7.01 (dd, *J* = 9.0, 3.0 Hz, 1H), 6.92–6.91 (d, *J* = 9.0 Hz, 1H), 3.80 (s, 3H), 3.73 (s, 3H), 3.48 (s, 2H), 2.87 – 2.80 (hept, *J* = 6.8 Hz, 1H), 1.23 – 1.22 (d, *J* = 6.9 Hz, 6H). ^{13}C NMR (101 MHz, $CDCl_3$) δ 162.5, 153.3, 151.9, 151.5, 141.9, 117.9, 116.1, 115.71, 112.5, 77.4, 56.0, 55.9, 36.2, 23.5, 19.4. HRMS ($^+$APCI) (*m/z*): [M + H$^+$] calcd for $C_{15}H_{19}O_2N_4{}^{32}S$, 319.1223; found, 319.1230. Purity: >95% (^{1}H NMR).

3-(2,5-dichloro)-6-isopropyl-7*H*-[1,2,4]triazolo[3,4-*b*][1,3,4]-thiadiazine (10e)

Following **GP3**, 4-amino-5-(2,5-dichlorophenyl)-4H-1, 2, 4-triazole-3-thiol (130 mg, 0.5 mmol) and 1-bromo-3-methylbutan-2-one (98 mg, 0.6 mmol) were refluxed in 10 mL absolute ethanol for 8 h. The reaction was then concentrated under vacuum and purified by flash column chromatography on Florisil® (MeOH/CH_2Cl_2 0:100 to 5:95), affording **10e** (141 mg, 87% yield) as a white solid (mp 188-191 ºC): ^{1}H NMR (600 MHz, Chloroform-*d*) δ 7.66 (t, *J* = 1.4 Hz, 1H), 7.43 (d, *J* = 1.4 Hz, 2H), 3.50 (s, 2H), 2.83 (hept, *J* = 6.8 Hz, 1H), 1.23 (d, *J* = 6.8 Hz, 6H). ^{13}C NMR (101 MHz, $CDCl_3$) δ 163.8, 132.9, 132.7, 132.3, 131.7, 131.2, 127.3, 36.4, 29.8, 29.5, 24.0, 19.4. HRMS ($^+$APCI) (*m/z*): [M + H$^+$] calcd for $C_{13}H_{13}N_4{}^{35}Cl_2{}^{32}S$, 327.0233; found, 327.0241. Purity: >95% (^{1}H NMR).

References

1. Han, S. T.; Rab, A.; Pellicore, M. J.; Davis, E. F.; McCague, A. F.; Evans, T. A.; Joynt, A. T.; Lu, Z.; Cai, Z.; Raraigh, K. S.; Hong, J. S.; Sheppard, D. N.; Sorscher, E. J.; Cutting, G. R., Residual function of cystic fibrosis mutants predicts response to small molecule CFTR modulators. *JCI Insight* **2018,** *3* (14).

2. Li, Z.; Liu, Y.; Bai, X.; Deng, Q.; Wang, J.; Zhang, G.; Xiao, C.; Mei, Y.; Wang, Y., SAR studies on 1,2,4-triazolo[3,4-b][1,3,4]thiadiazoles as inhibitors of Mtb shikimate dehydrogenase for the development of novel antitubercular agents. *RSC Advances* **2015,** *5* (118), 97089-97101.

3. Li, Z.; Bai, X.; Deng, Q.; Zhang, G.; Zhou, L.; Liu, Y.; Wang, J.; Wang, Y., Preliminary SAR and biological evaluation of antitubercular triazolothiadiazine derivatives against drug-susceptible and drug-resistant Mtb strains. *Biorg. Med. Chem.* **2017,** *25* (1), 213-220.

¹H NMR Spectra

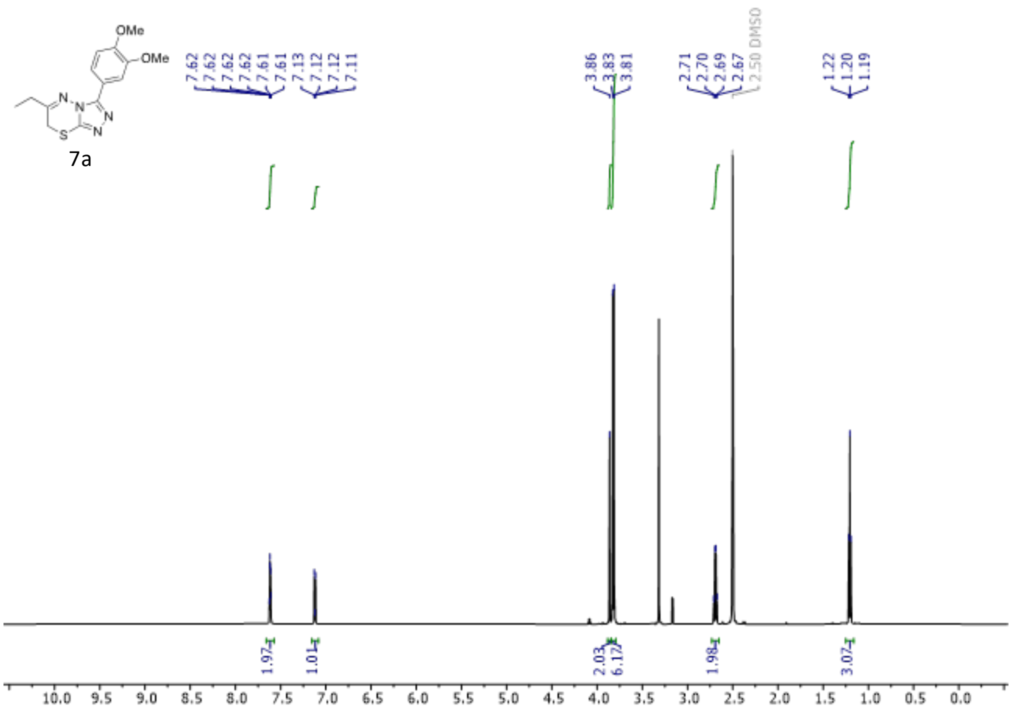

7a

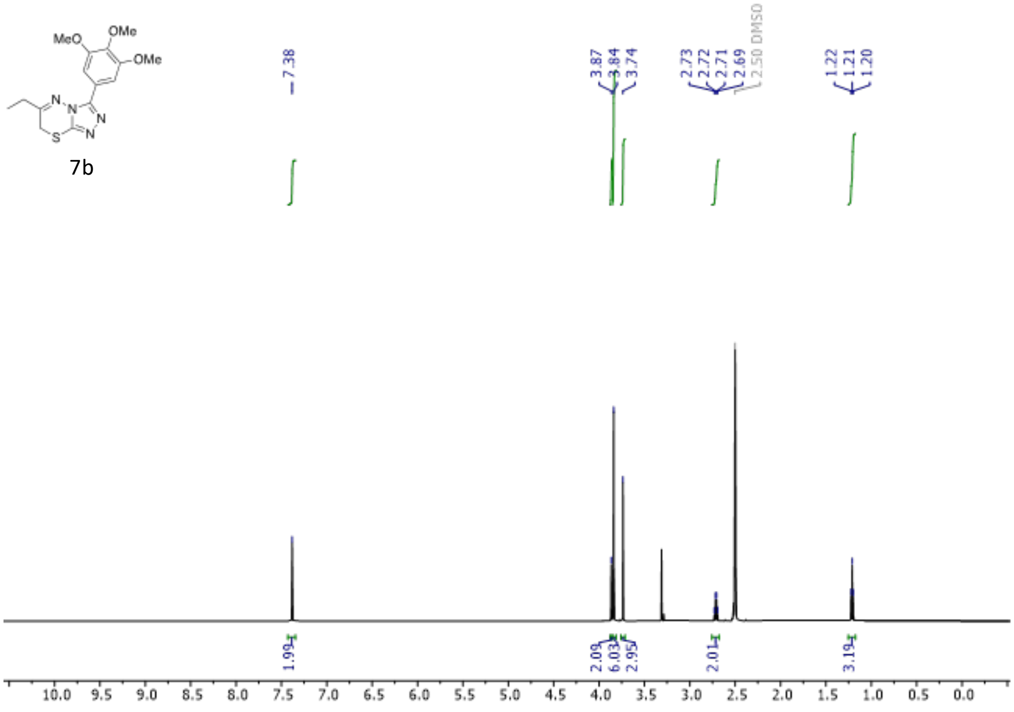

7b

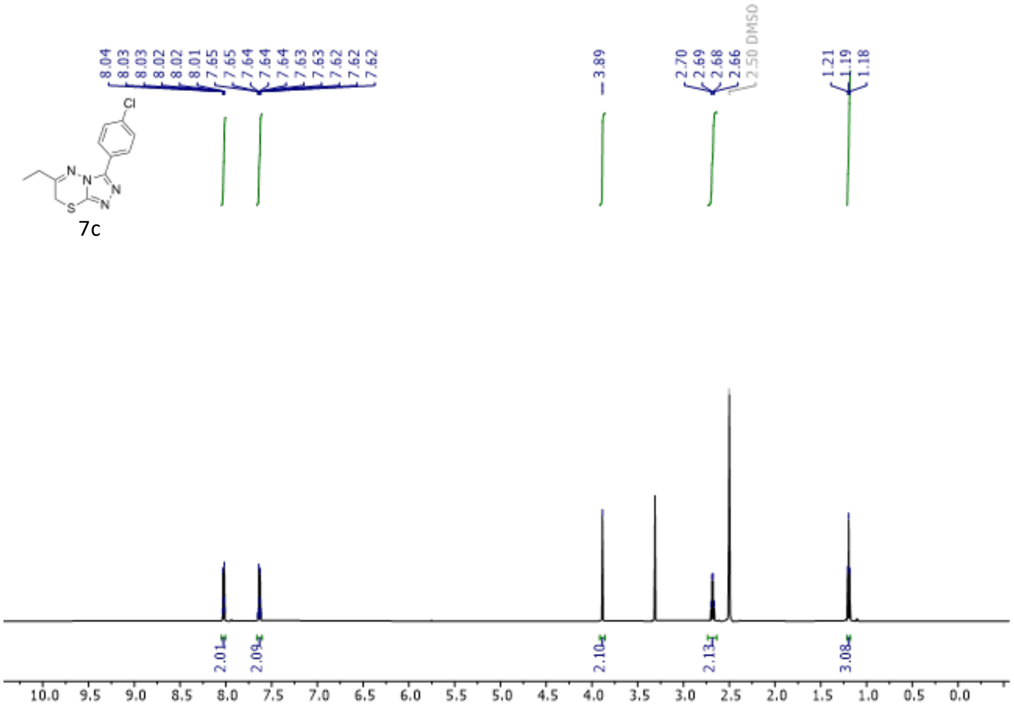

7c

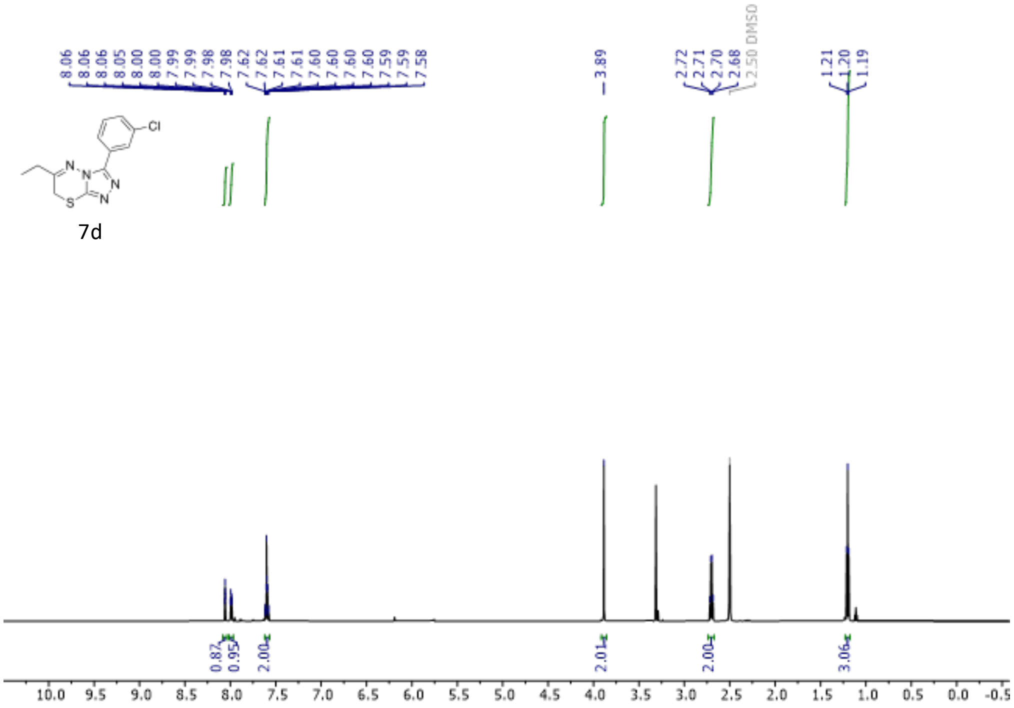

7d

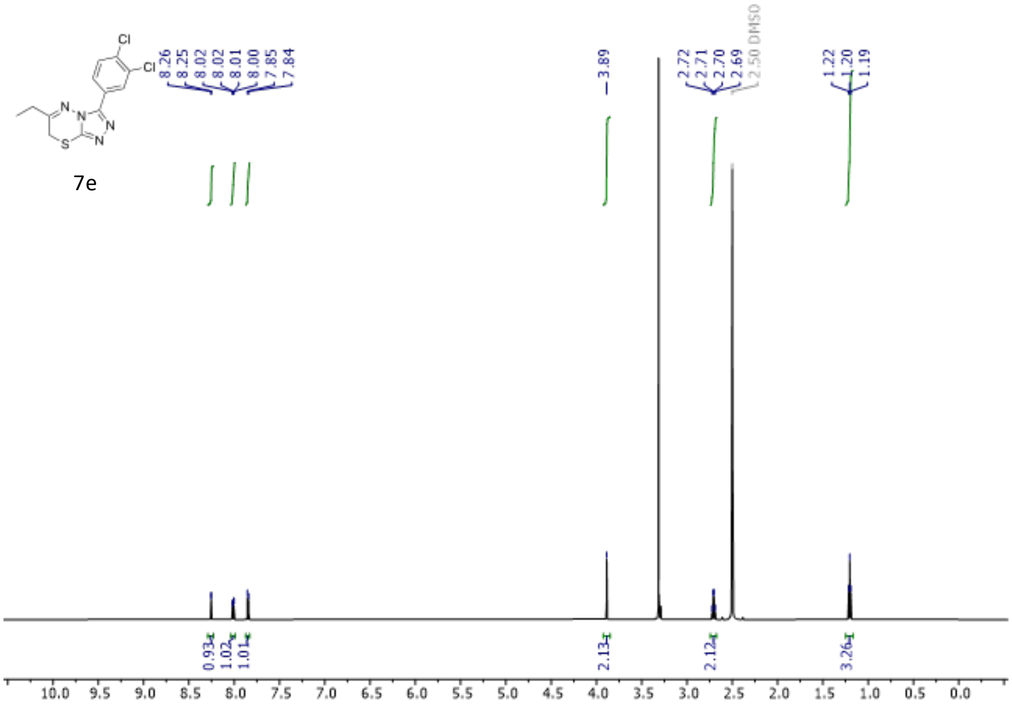
7e
8.26
8.25
8.02
8.02
8.01
8.00
7.85
7.84
3.89
2.72
2.71
2.70
2.69
2.50 DMSO
1.22
1.20
1.19
0.93
1.02
1.01
2.13
2.12
3.26
10.0 9.5 9.0 8.5 8.0 7.5 7.0 6.5 6.0 5.5 5.0 4.5 4.0 3.5 3.0 2.5 2.0 1.5 1.0 0.5 0.0

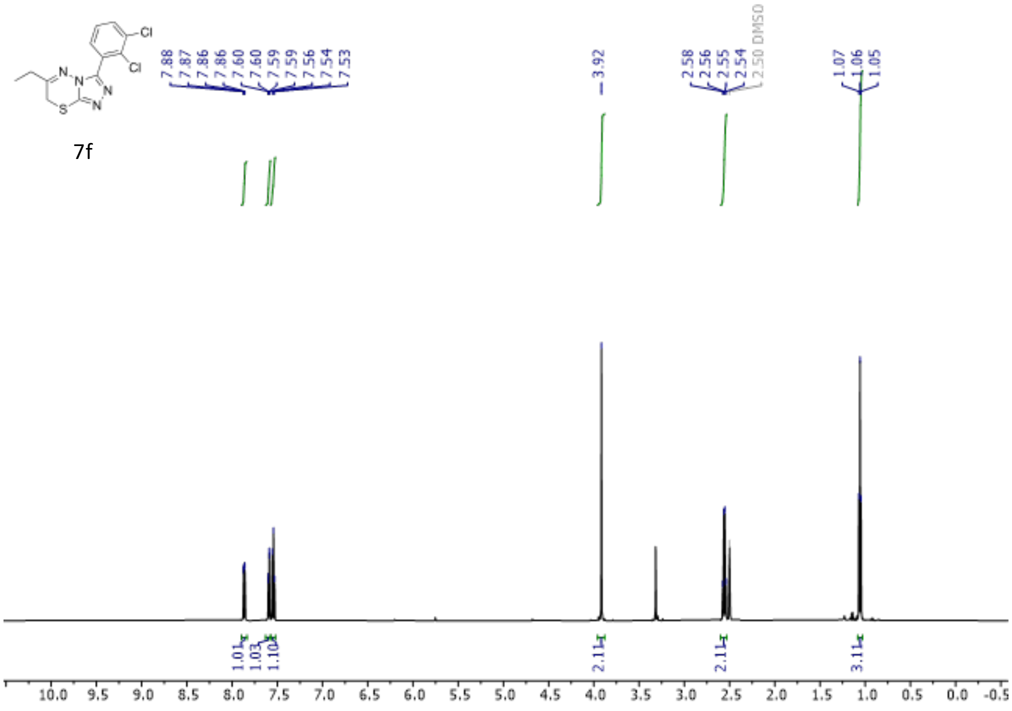
7f
7.88
7.87
7.86
7.86
7.60
7.60
7.59
7.59
7.56
7.54
7.53
3.92
2.58
2.56
2.55
2.54
2.50 DMSO
1.07
1.06
1.05
1.01
1.03
1.10
2.11
2.11
3.11
10.0 9.5 9.0 8.5 8.0 7.5 7.0 6.5 6.0 5.5 5.0 4.5 4.0 3.5 3.0 2.5 2.0 1.5 1.0 0.5 0.0 -0.5

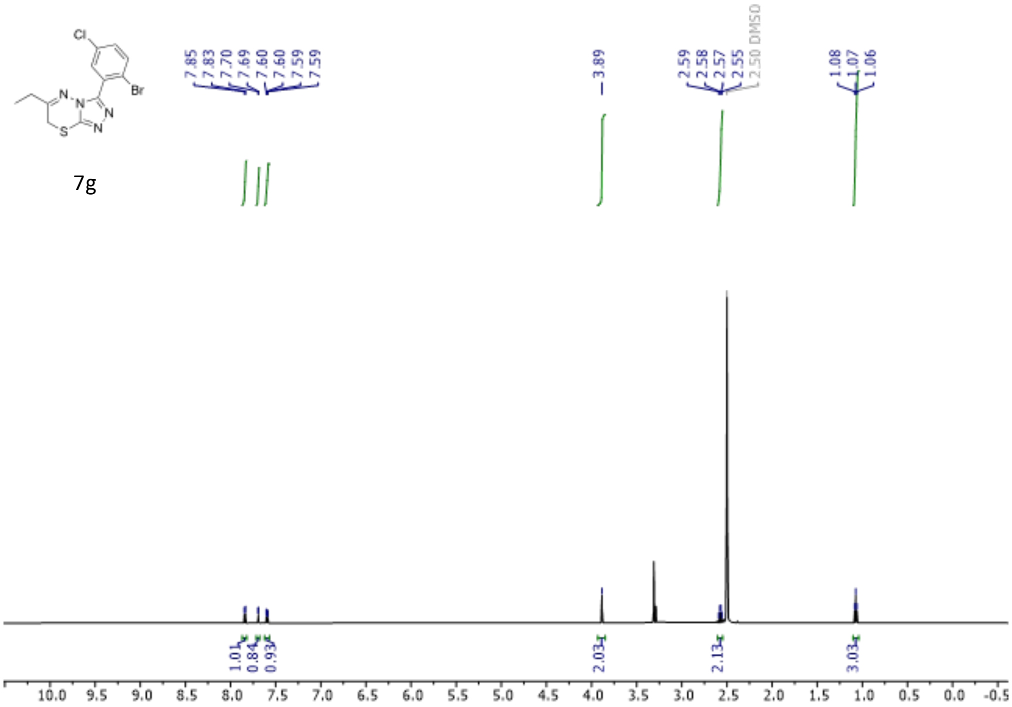

7g

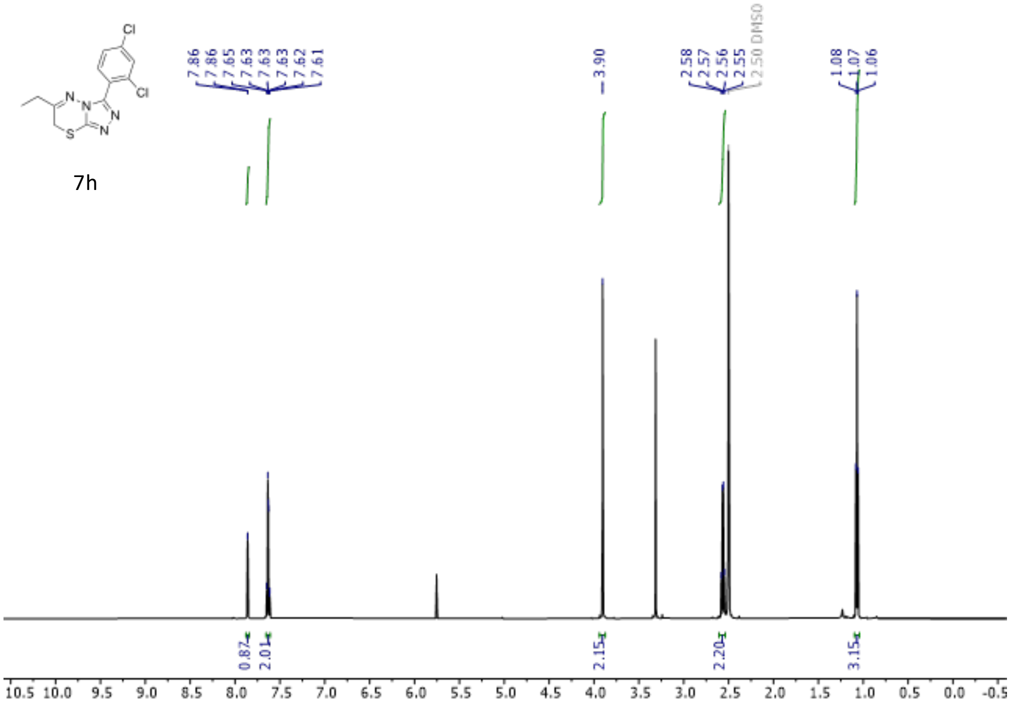

7h

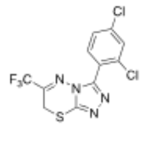
Cl
Cl
F3C
N
N
N
N
S

8a

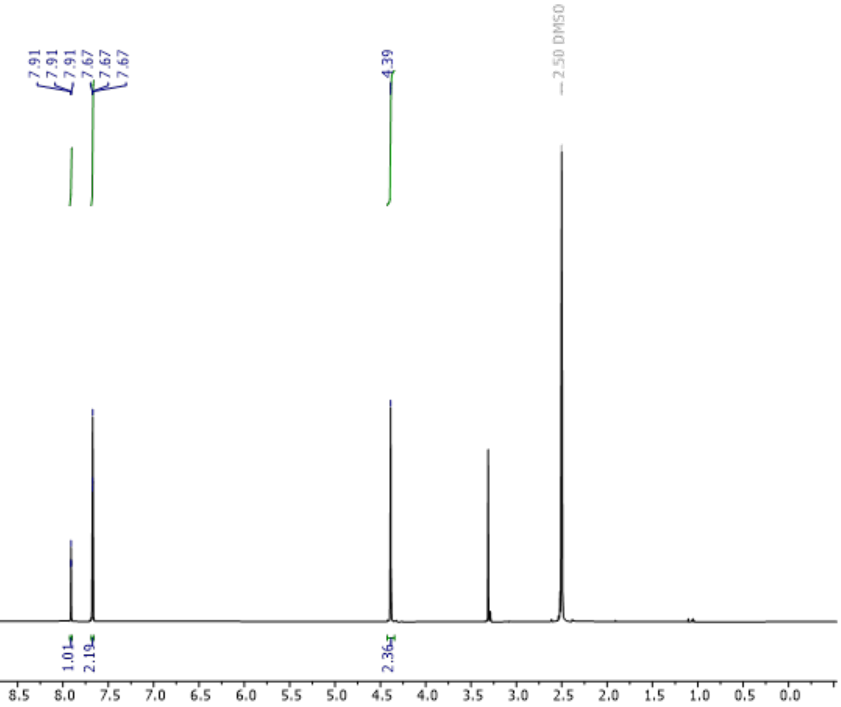
7.91
7.91
7.91
7.67
7.67
7.67
4.39
2.50 DMSO
1.01
2.19
2.36
10.0 9.5 9.0 8.5 8.0 7.5 7.0 6.5 6.0 5.5 5.0 4.5 4.0 3.5 3.0 2.5 2.0 1.5 1.0 0.5 0.0

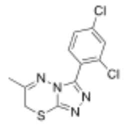
Cl
Cl
N
N
N
N
S

8b

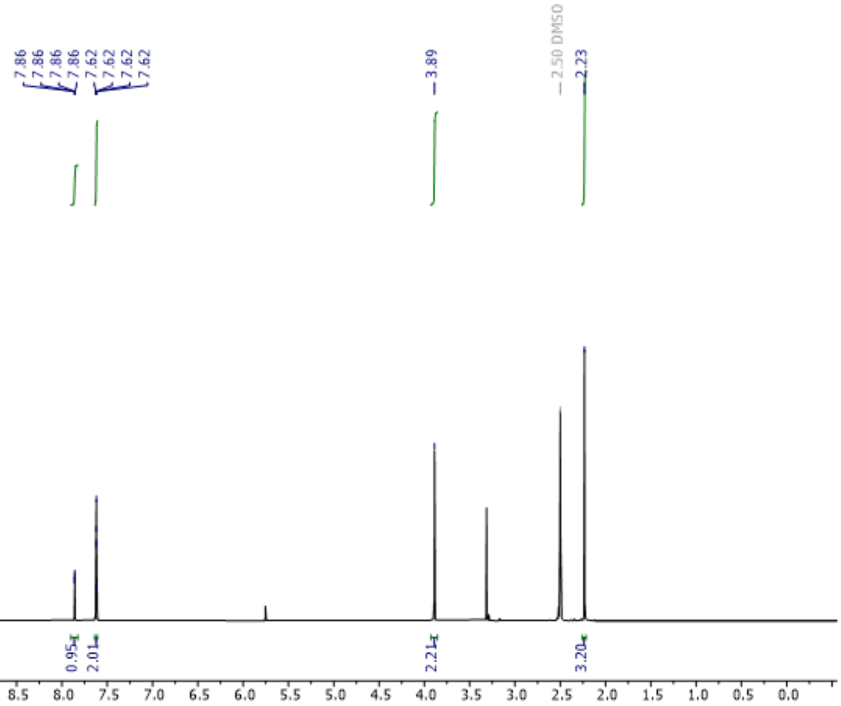
7.86
7.86
7.86
7.86
7.62
7.62
7.62
7.62
3.89
2.50 DMSO
2.23
0.95
2.01
2.21
3.20
10.0 9.5 9.0 8.5 8.0 7.5 7.0 6.5 6.0 5.5 5.0 4.5 4.0 3.5 3.0 2.5 2.0 1.5 1.0 0.5 0.0

8c

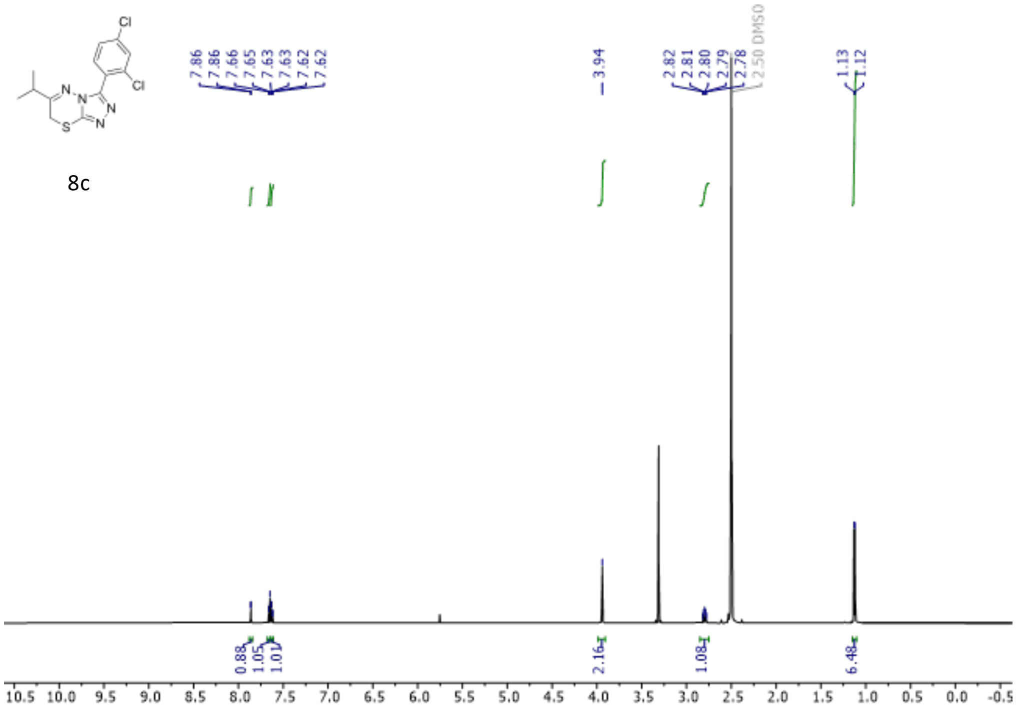
Cl
Cl
N
N
N
N
S
7.86
7.86
7.66
7.65
7.63
7.63
7.62
7.62
3.94
2.82
2.81
2.80
2.79
2.78
2.50 DMSO
1.13
1.12
0.88
1.05
1.01
2.16
1.08
6.48
10.5 10.0 9.5 9.0 8.5 8.0 7.5 7.0 6.5 6.0 5.5 5.0 4.5 4.0 3.5 3.0 2.5 2.0 1.5 1.0 0.5 0.0 -0.5

8d

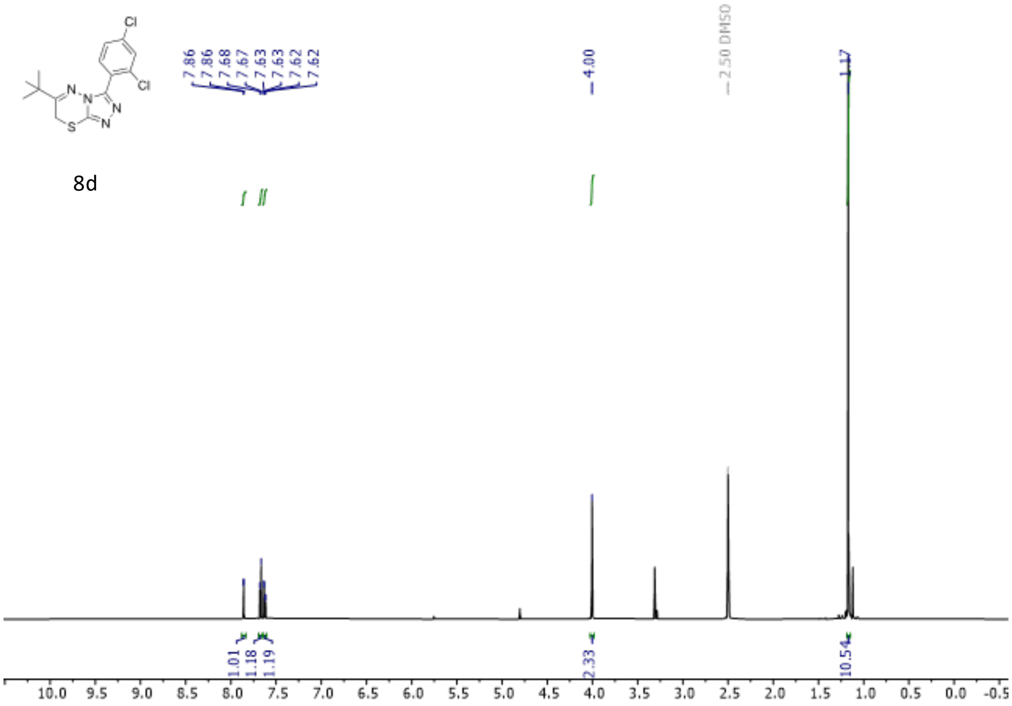
Cl
Cl
N
N
N
N
S
7.86
7.86
7.68
7.67
7.63
7.63
7.62
7.62
4.00
2.50 DMSO
1.17
1.01
1.18
1.19
2.33
10.54
10.0 9.5 9.0 8.5 8.0 7.5 7.0 6.5 6.0 5.5 5.0 4.5 4.0 3.5 3.0 2.5 2.0 1.5 1.0 0.5 0.0 -0.5

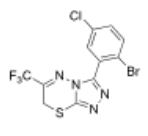

Cl
F_3C
N
N
N
N
S
Br

9a

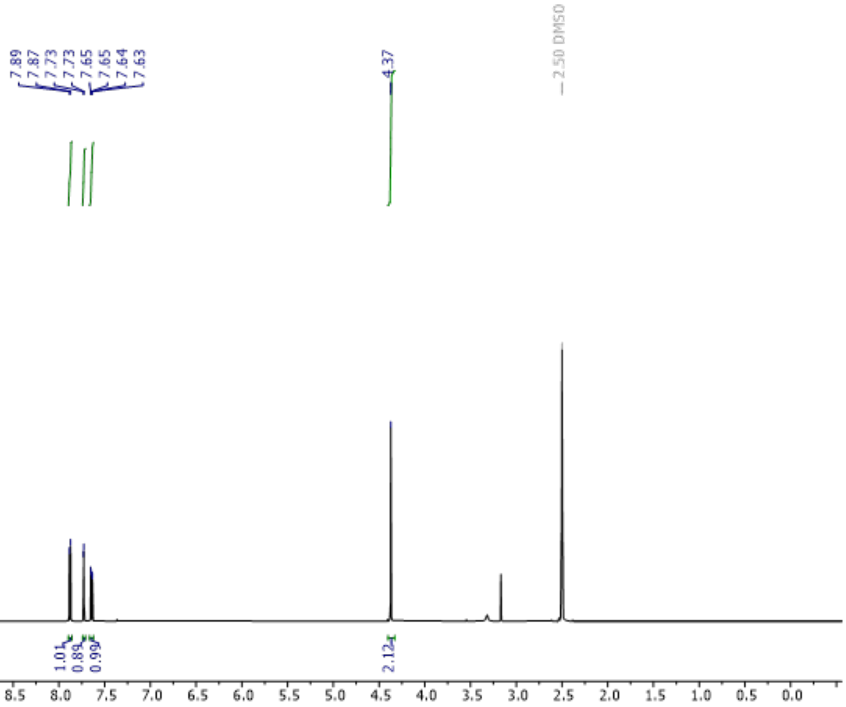

7.89
7.87
7.73
7.73
7.65
7.65
7.64
7.63
4.37
2.50 DMSO
1.01
0.89
0.99
2.12
10.0 9.5 9.0 8.5 8.0 7.5 7.0 6.5 6.0 5.5 5.0 4.5 4.0 3.5 3.0 2.5 2.0 1.5 1.0 0.5 0.0

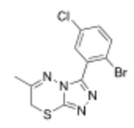

Cl
N
N
N
N
S
Br

9b

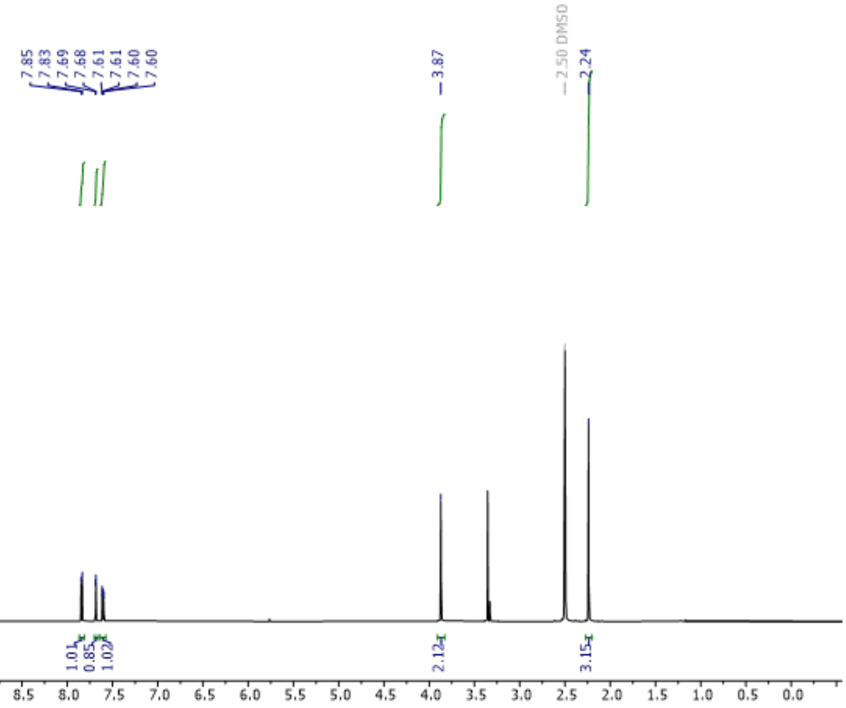

7.85
7.83
7.69
7.68
7.61
7.61
7.60
7.60
3.87
2.50 DMSO
2.24
1.01
0.85
1.02
2.12
3.15
10.0 9.5 9.0 8.5 8.0 7.5 7.0 6.5 6.0 5.5 5.0 4.5 4.0 3.5 3.0 2.5 2.0 1.5 1.0 0.5 0.0

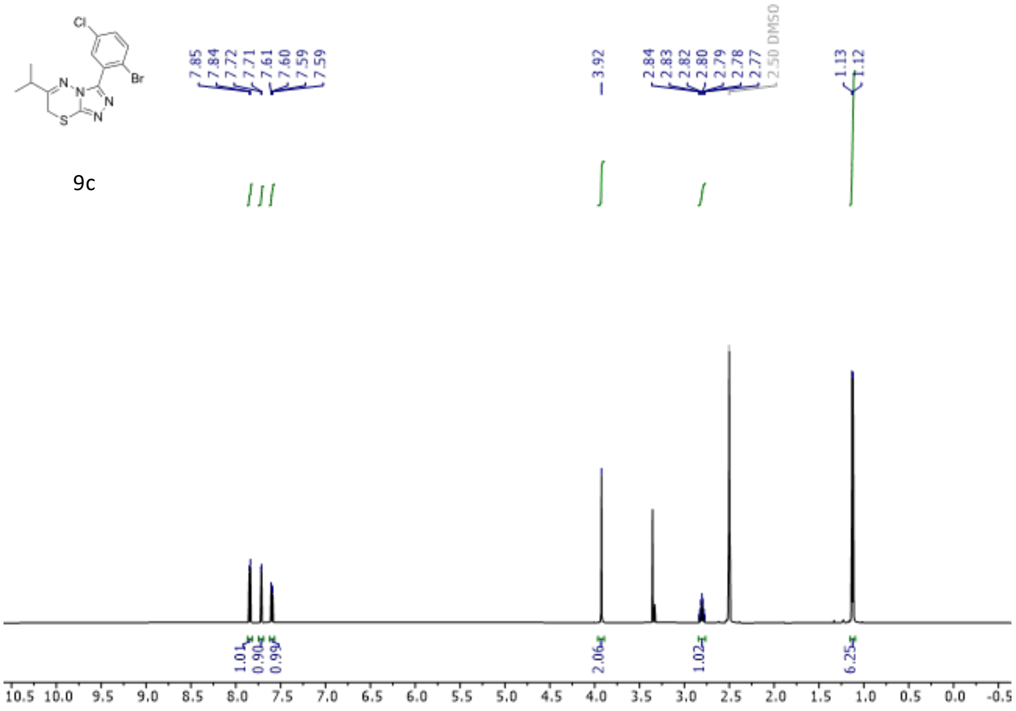

9c

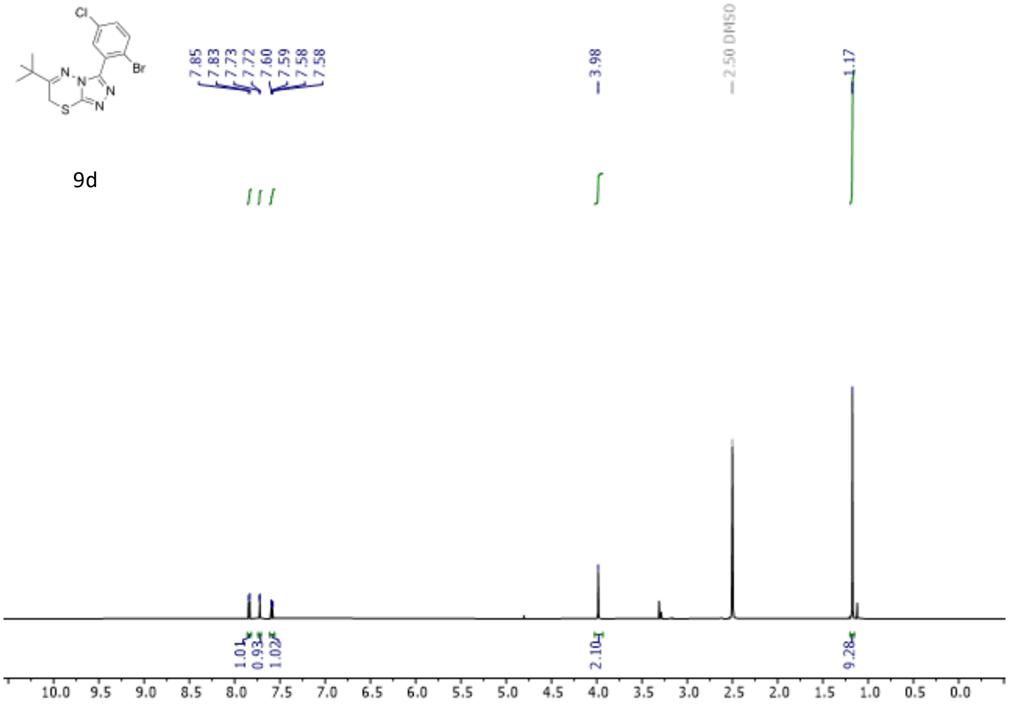

9d

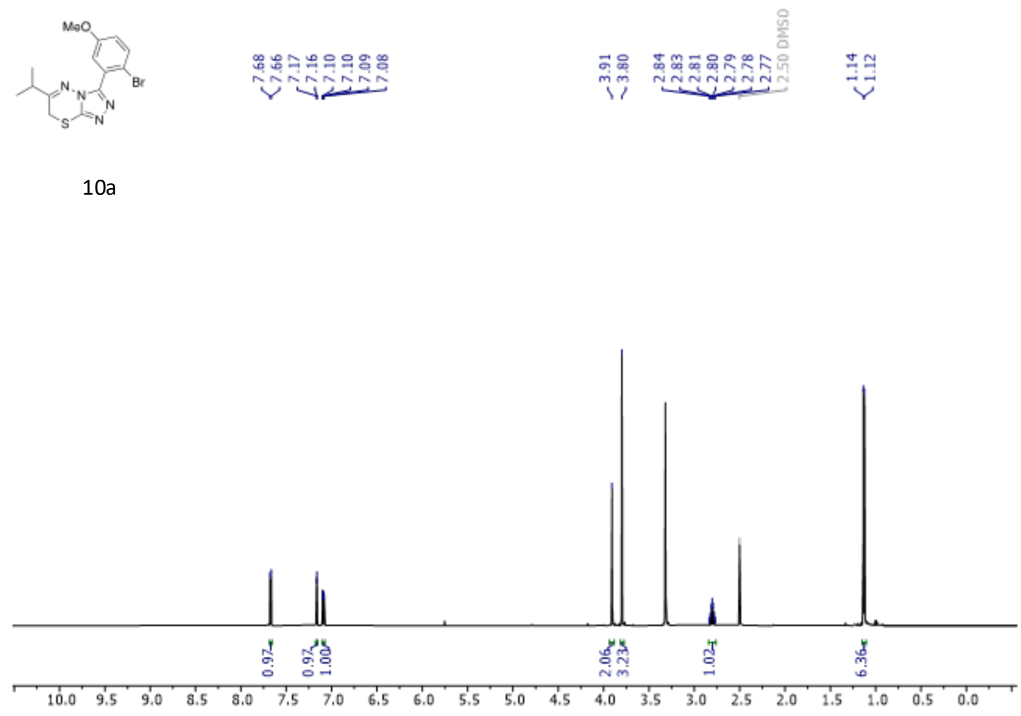

10a

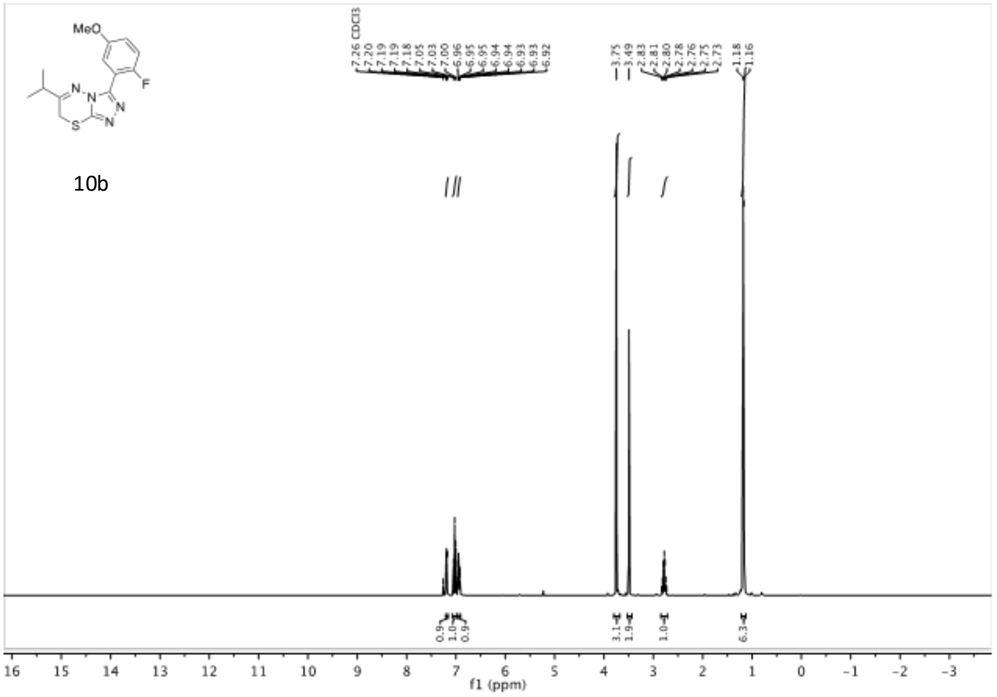

10b

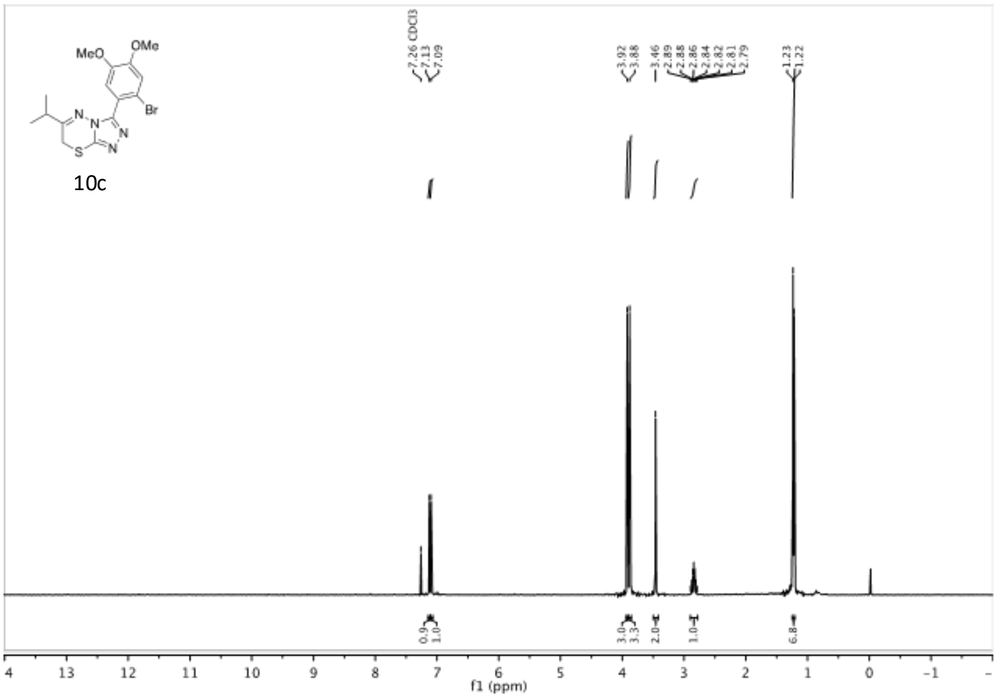
10c
f1 (ppm)

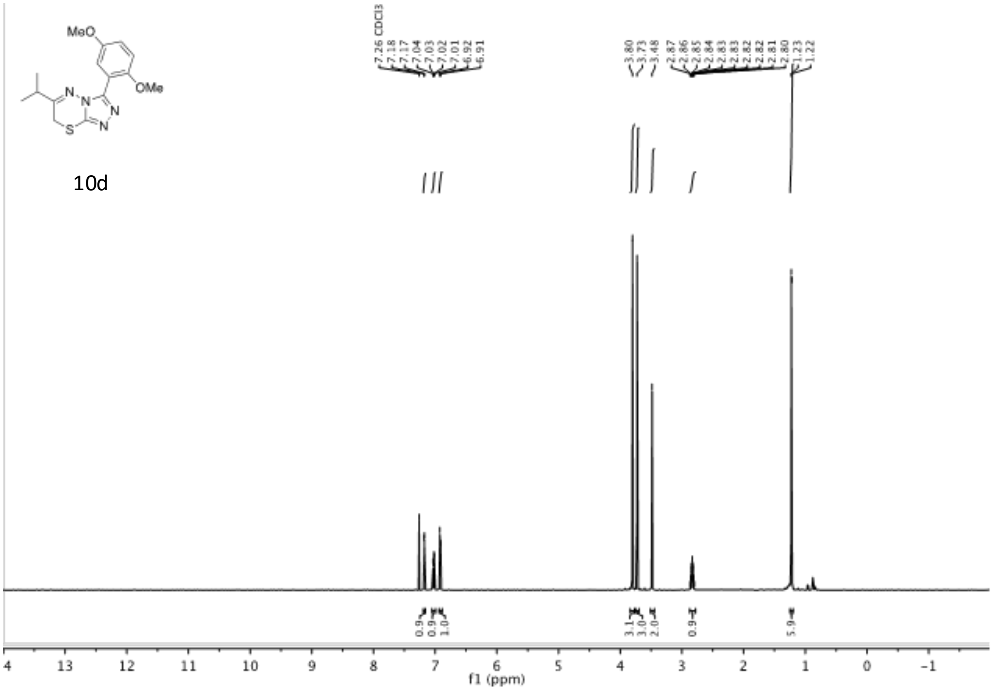
10d
f1 (ppm)

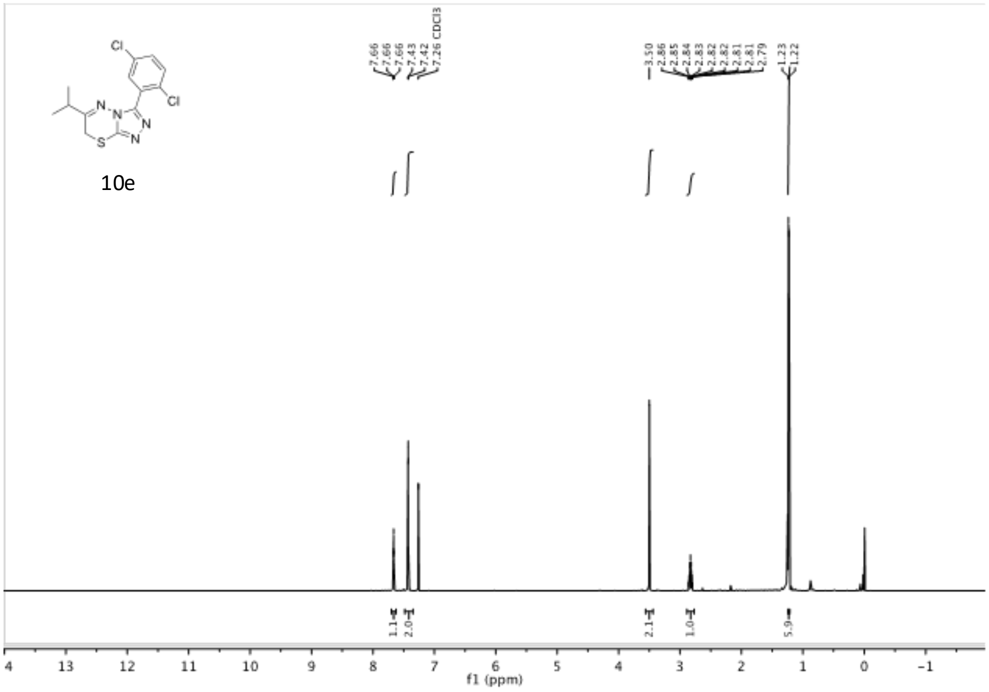

^{13}C NMR Spectra

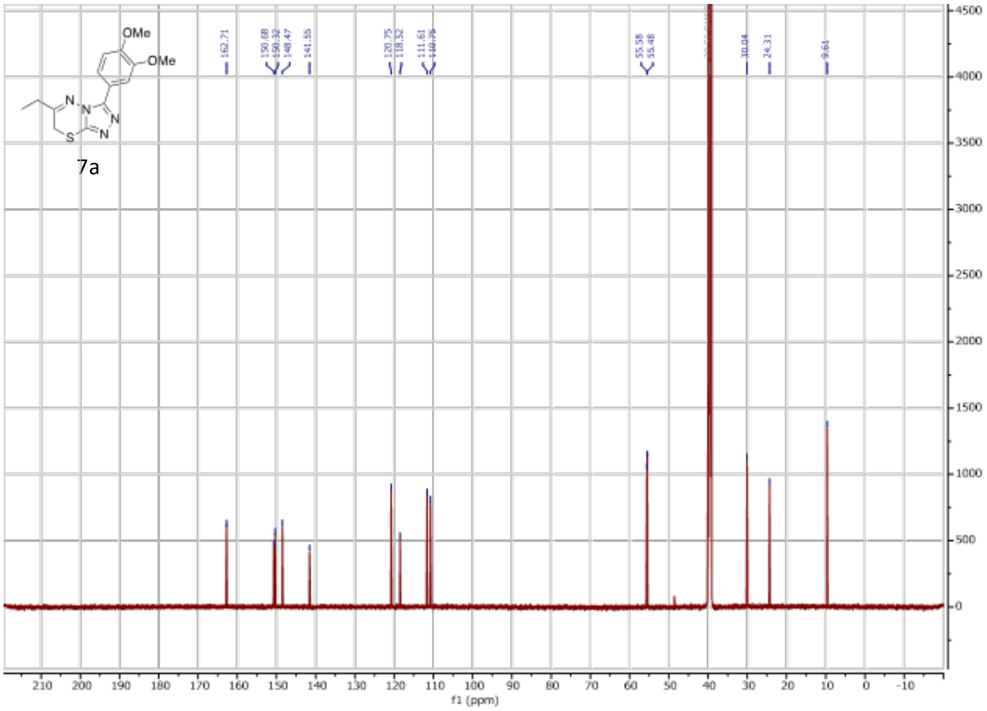

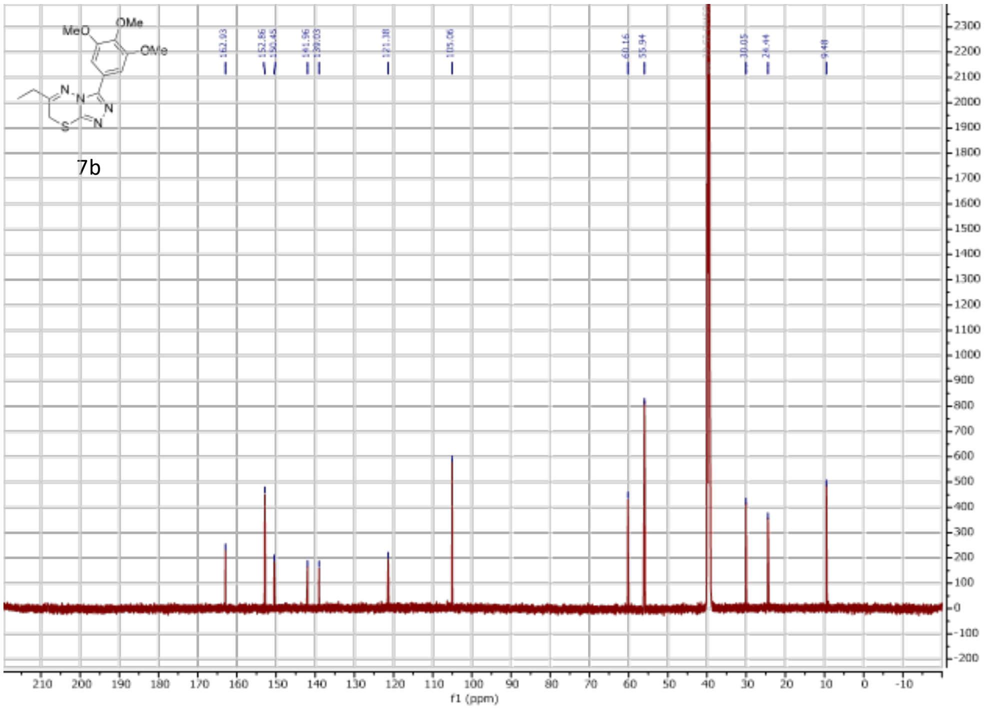

OMe
MeO
OMe
N
N
N
N
S
162.93
152.86
150.45
141.96
139.03
121.38
105.06
60.16
55.94
30.05
24.44
9.48
2300
2200
2100
2000
1900
1800
1700
1600
1500
1400
1300
1200
1100
1000
900
800
700
600
500
400
300
200
100
0
-100
-200
210 200 190 180 170 160 150 140 130 120 110 100 90 80 70 60 50 40 30 20 10 0 -10
f1 (ppm)

7b

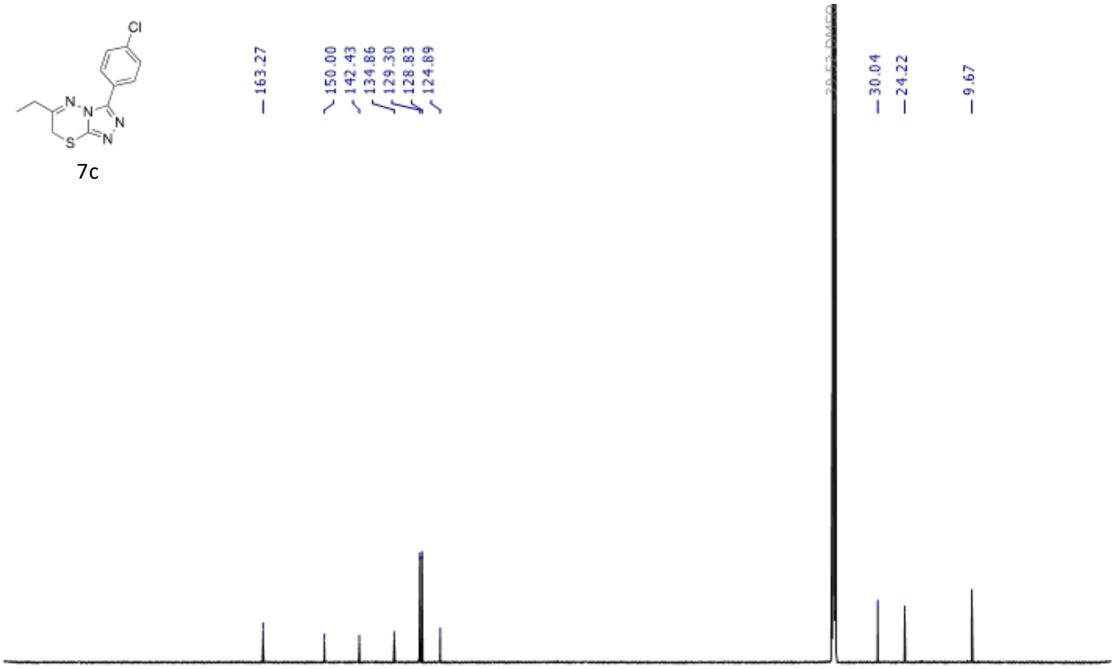

Cl
N
N
N
N
S
163.27
150.00
142.43
134.86
129.30
128.83
124.89
30.04
24.22
9.67

7c

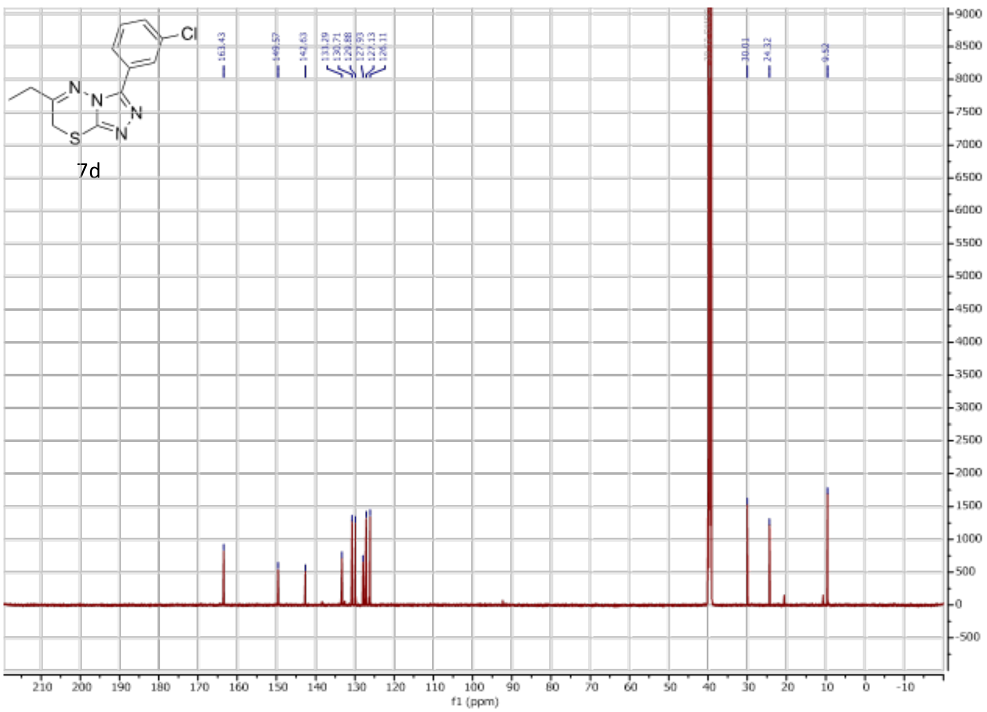
7d
163.43
149.57
142.63
133.29
130.71
129.88
127.93
127.13
126.11
30.01
24.32
9.52
f1 (ppm)

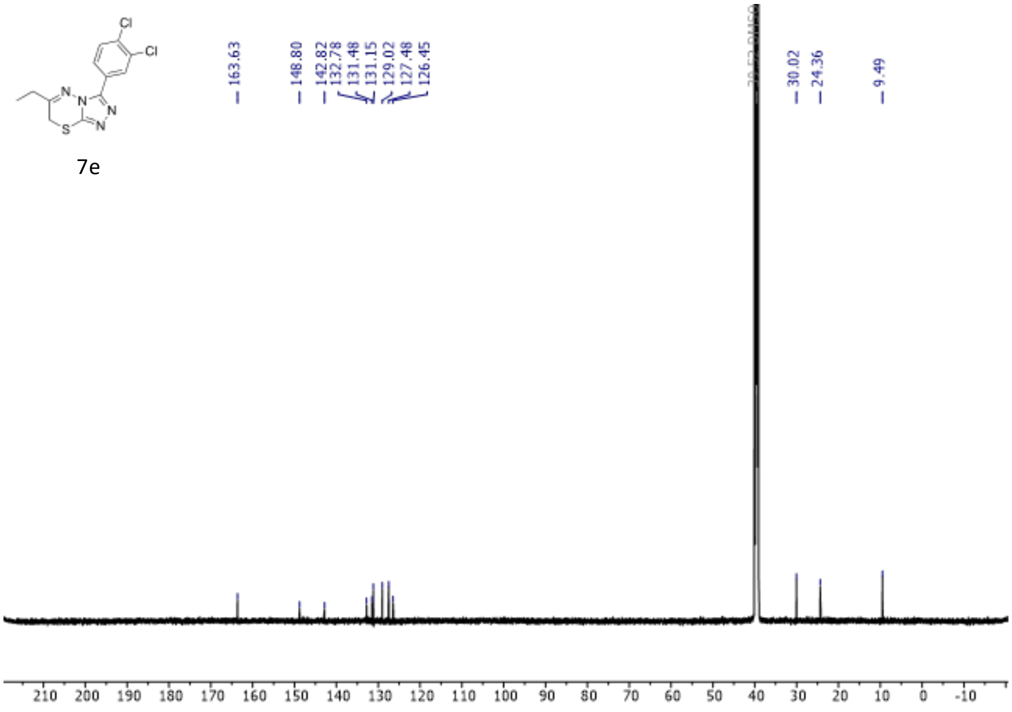
7e
163.63
148.80
142.82
132.78
131.48
131.15
129.02
127.48
126.45
30.02
24.36
9.49

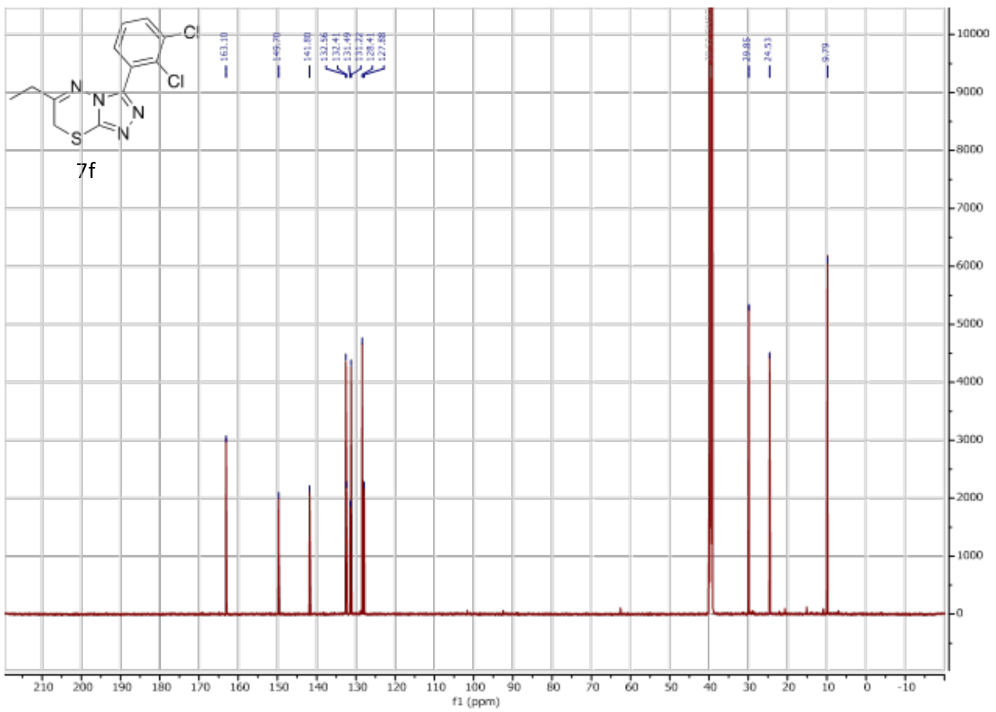
Cl
Cl
N
N
N
N
S
7f
163.10
149.70
141.80
132.56
132.41
131.49
131.22
128.41
127.88
29.85
24.53
9.79
f1 (ppm)

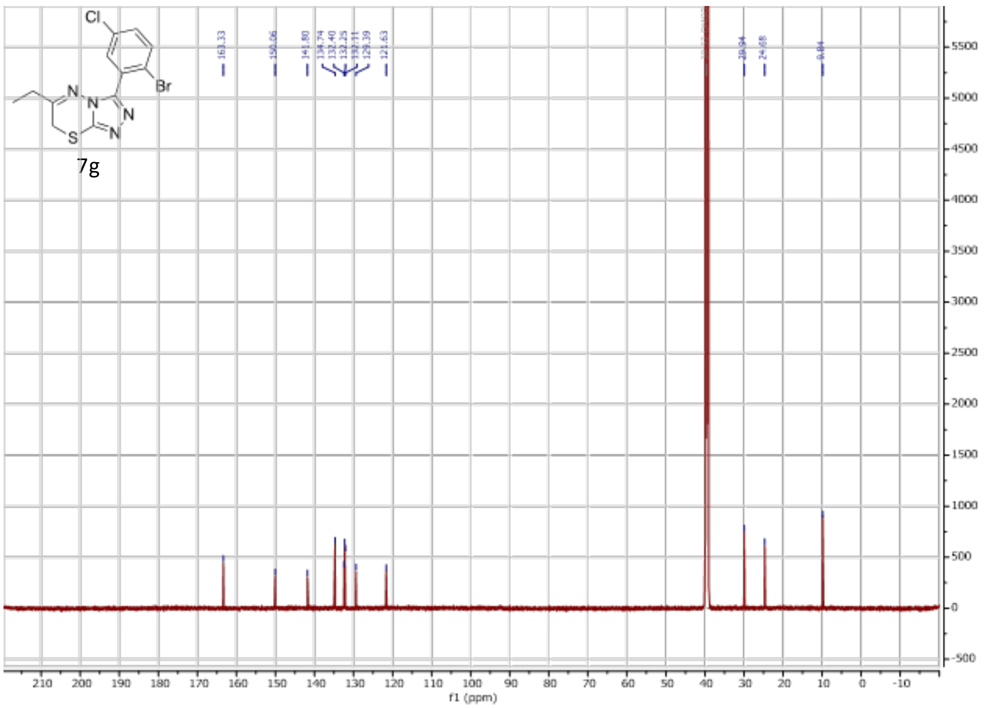
Cl
Br
N
N
N
N
S
7g
163.33
150.06
141.80
134.74
132.40
132.25
132.11
129.39
121.63
29.94
24.68
9.84
f1 (ppm)

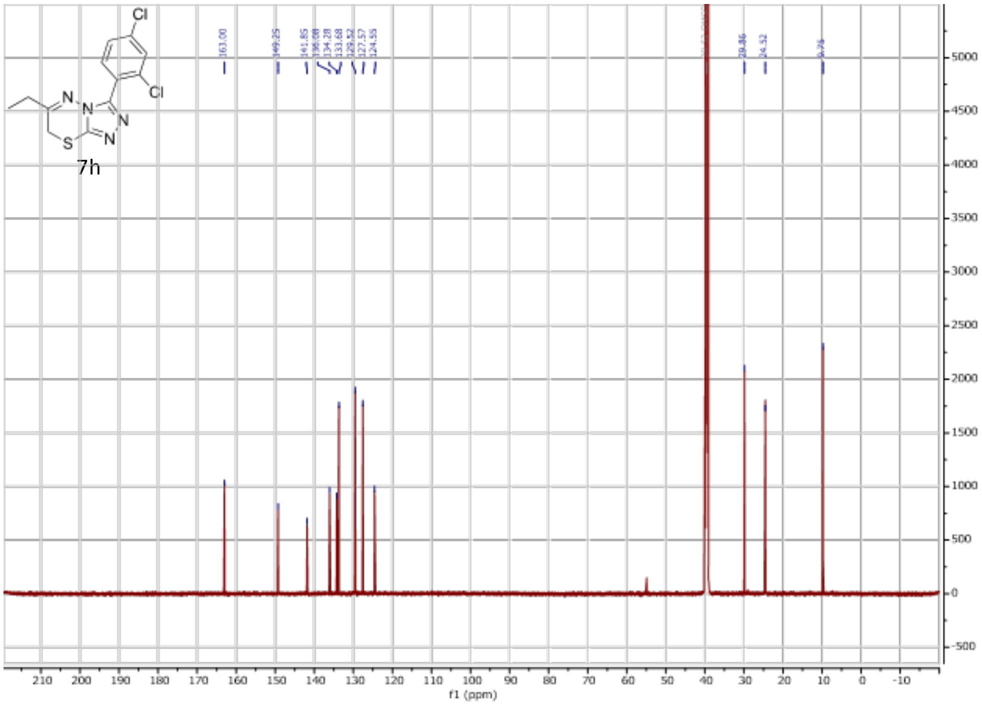

7h
f1 (ppm)

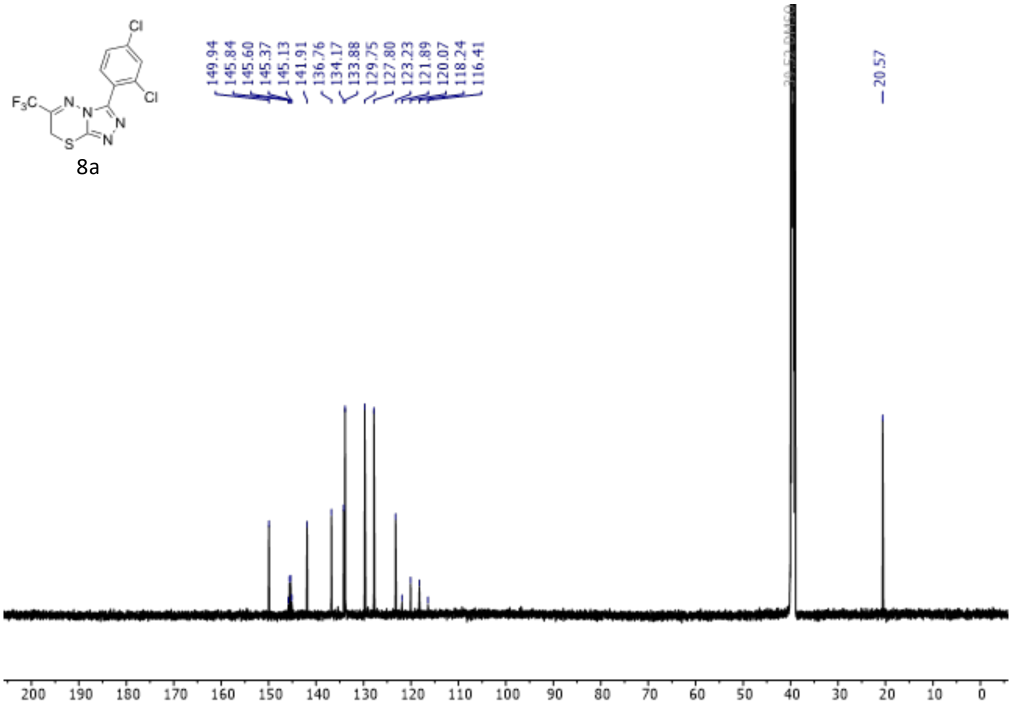

8a
149.94
145.84
145.60
145.37
145.13
141.91
136.76
134.17
133.88
129.75
127.80
123.23
121.89
120.07
118.24
116.41
20.57

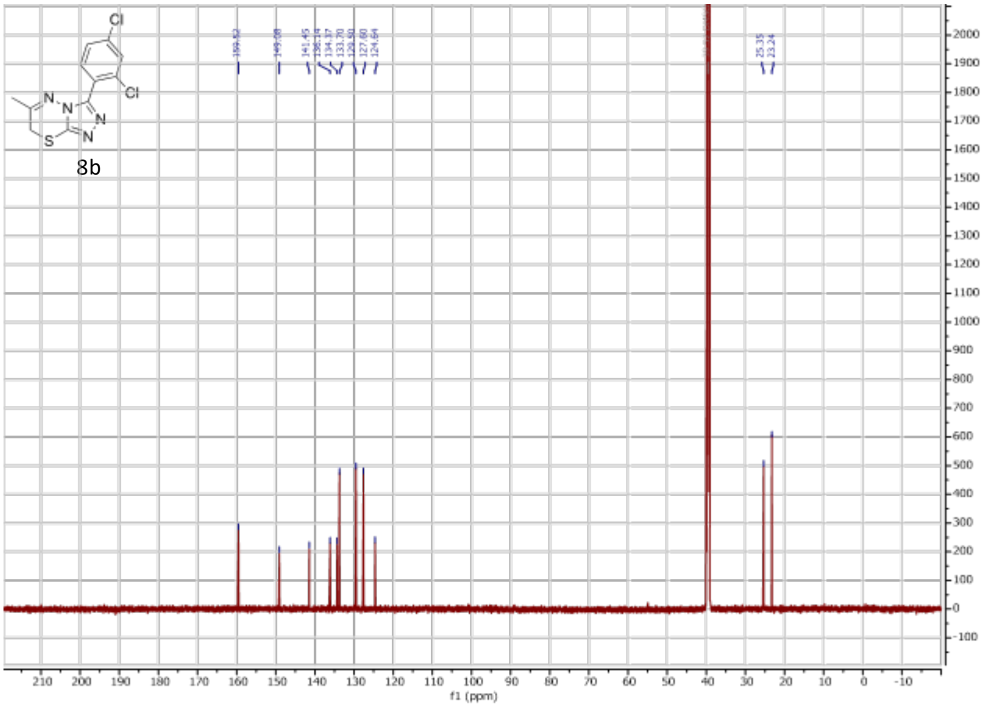
8b
Cl
Cl
N
N
S
141.45
136.14
134.37
133.70
129.90
127.60
124.64
25.35
23.24
f1 (ppm)

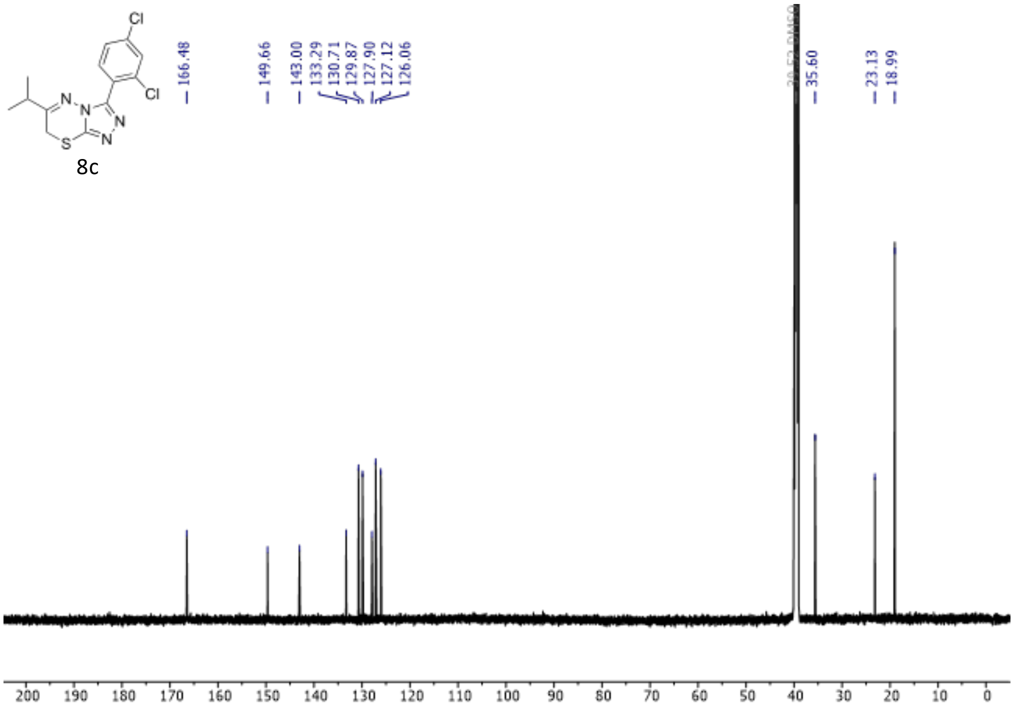
8c
Cl
Cl
N
N
S
166.48
149.66
143.00
133.29
130.71
129.87
127.90
127.12
126.06
35.60
23.13
18.99

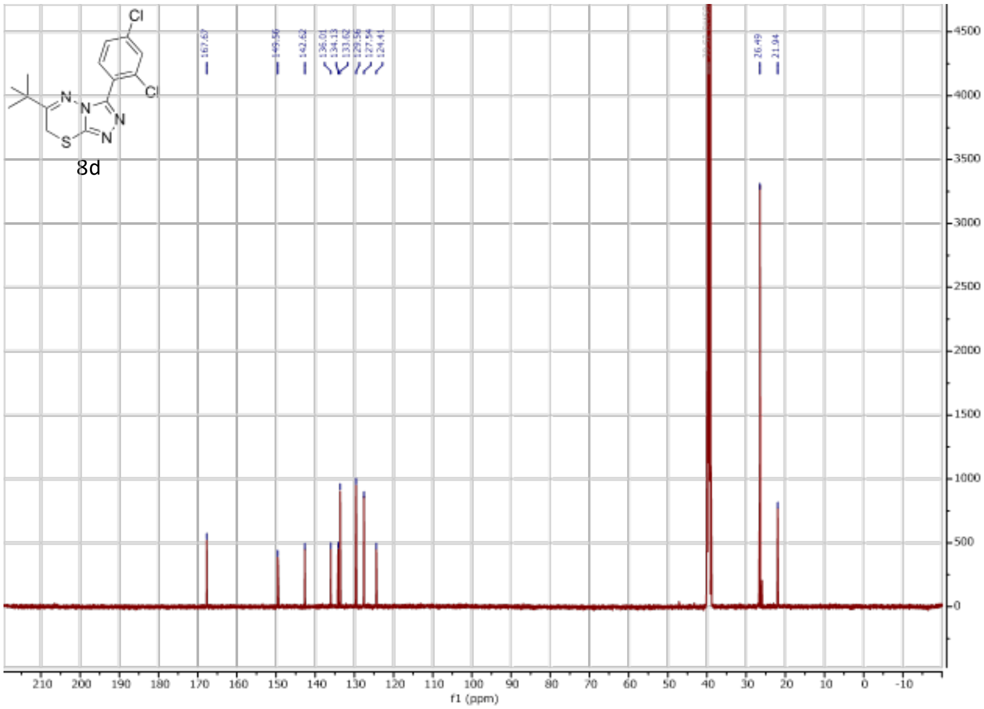
8d
167.67
149.56
142.62
136.01
134.13
133.62
129.56
127.54
124.41
26.49
21.94
f1 (ppm)

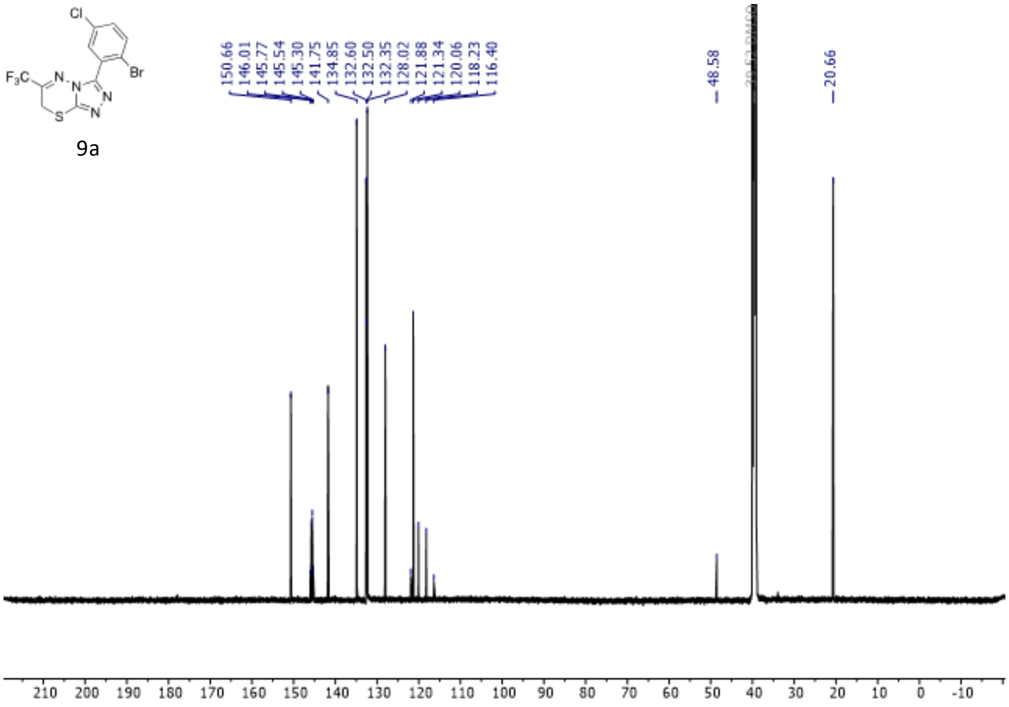
9a
150.66
146.01
145.77
145.54
145.30
141.75
134.85
132.60
132.50
132.35
128.02
121.88
121.34
120.06
118.23
116.40
48.58
20.66

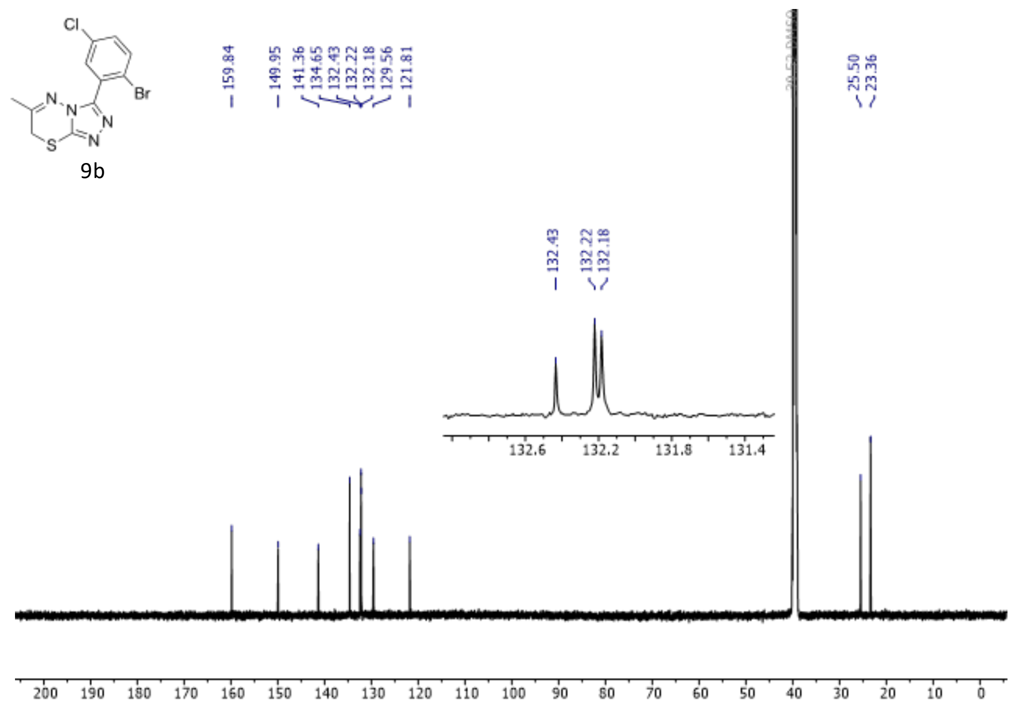

Cl
Br
N
S
159.84
149.95
141.36
134.65
132.43
132.22
132.18
129.56
121.81
25.50
23.36
132.6
132.2
131.8
131.4

9b

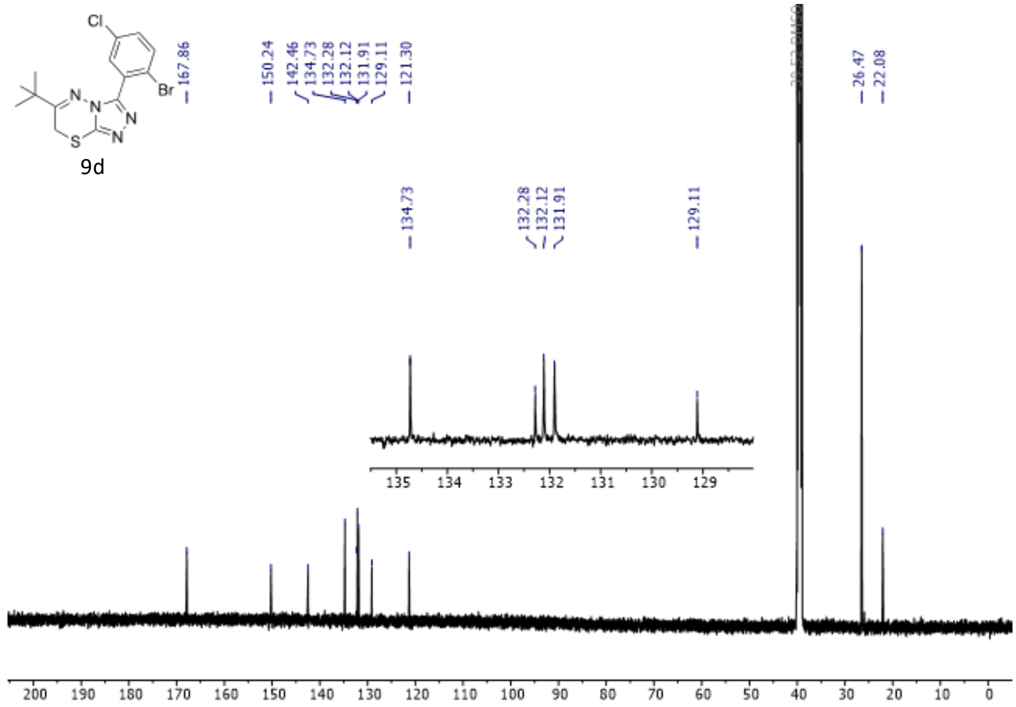

Cl
Br
N
S
167.86
150.24
142.46
134.73
132.28
132.12
131.91
129.11
121.30
26.47
22.08
135
134
133
132
131
130
129

9d

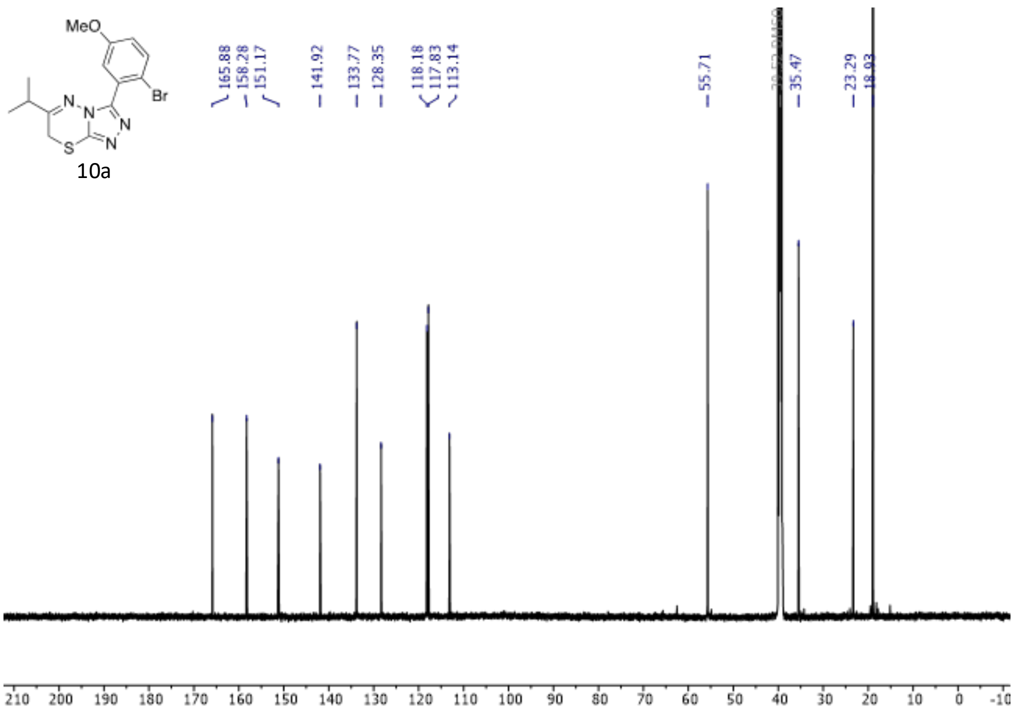

10a

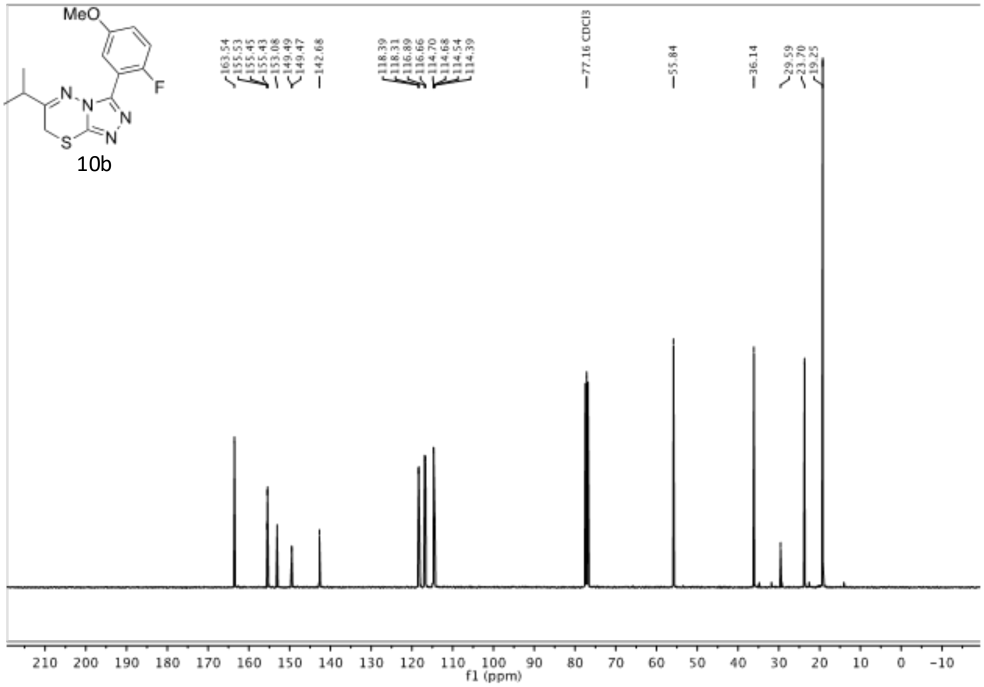

10b

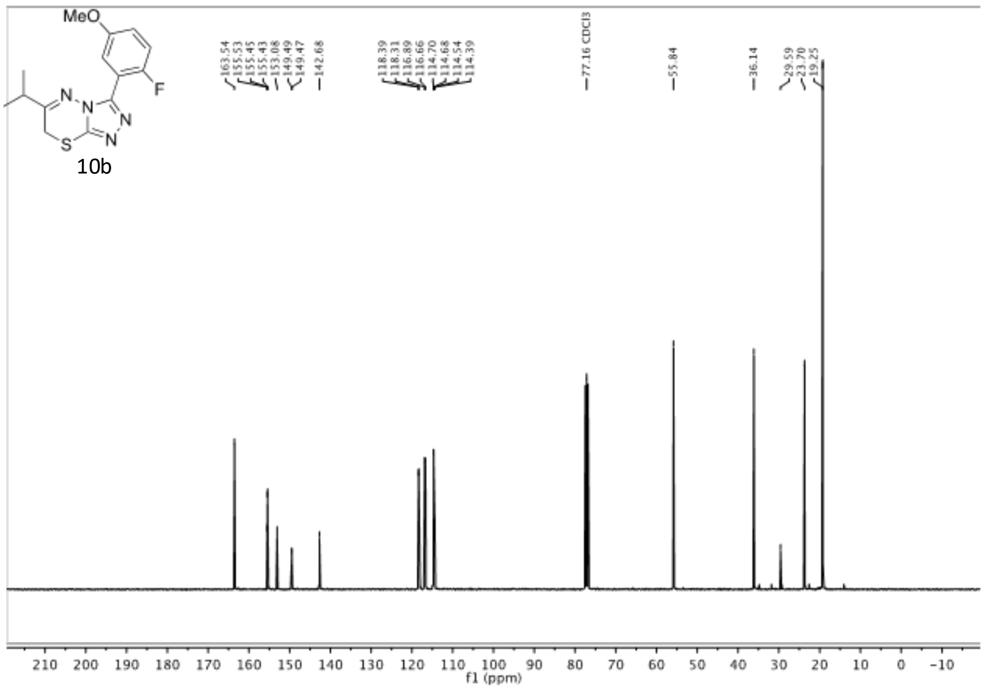
MeO
F
N
S
10b
163.54
155.53
155.45
155.43
153.08
149.49
149.47
142.68
118.39
118.31
116.89
116.66
114.70
114.68
114.54
114.39
77.16 CDCl3
55.84
36.14
29.59
23.70
19.25
210 200 190 180 170 160 150 140 130 120 110 100 90 80 70 60 50 40 30 20 10 0 -10
f1 (ppm)

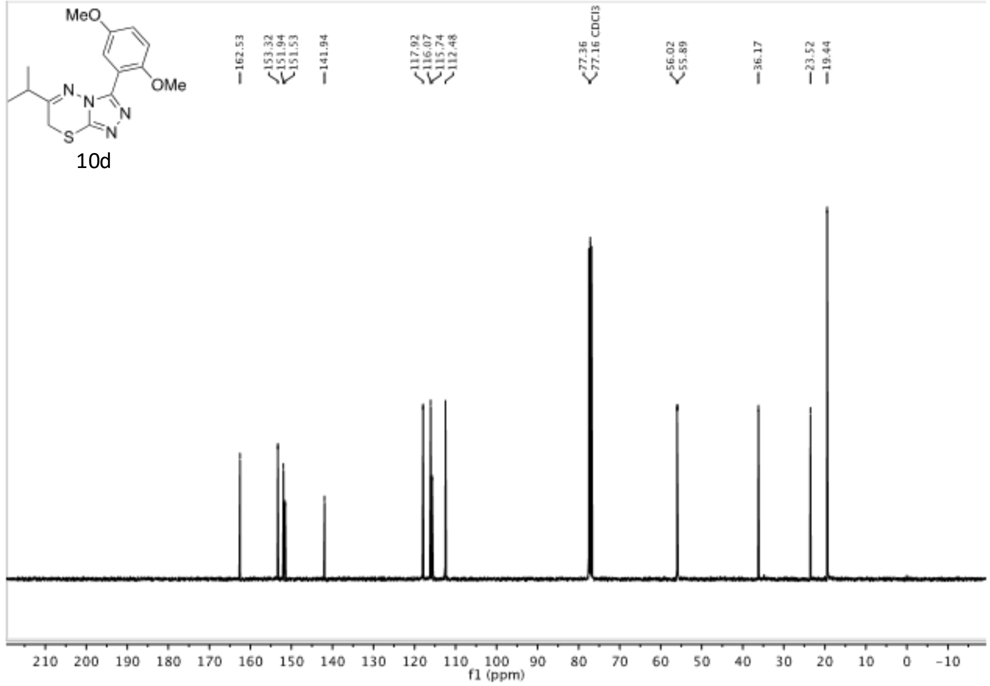
MeO
OMe
N
S
10d
162.53
153.32
151.94
151.53
141.94
117.92
116.07
115.74
112.48
77.36
77.16 CDCl3
56.02
55.89
36.17
23.52
19.44
210 200 190 180 170 160 150 140 130 120 110 100 90 80 70 60 50 40 30 20 10 0 -10
f1 (ppm)

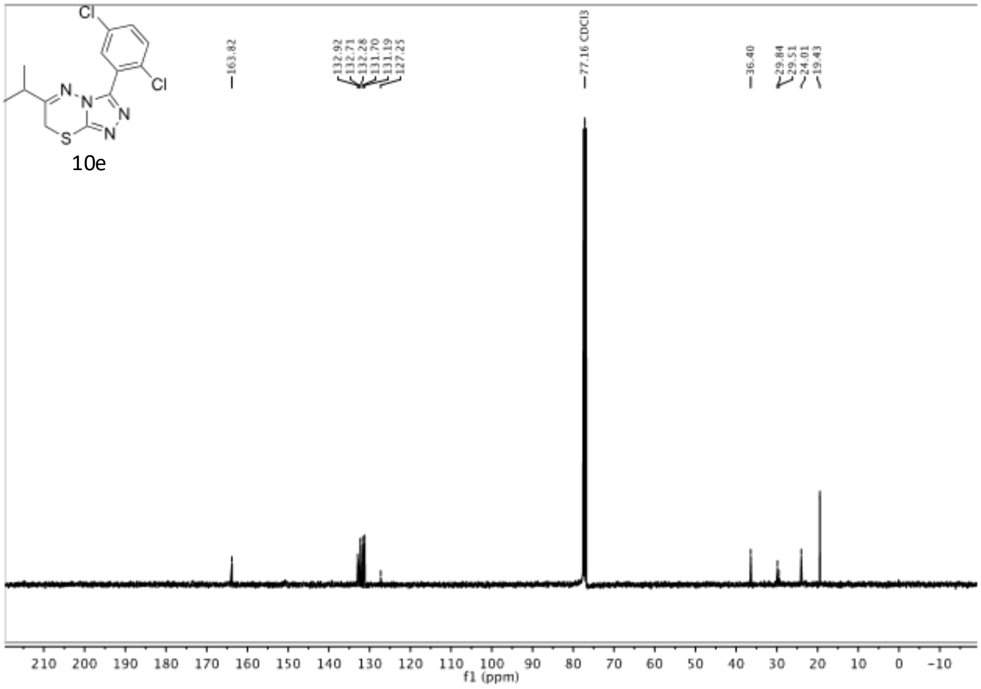

Cl
Cl
N
S
10e
163.82
132.92
132.71
132.28
131.70
131.19
127.25
77.16 CDCl3
36.40
29.84
29.51
24.01
19.43
210 200 190 180 170 160 150 140 130 120 110 100 90 80 70 60 50 40 30 20 10 0 -10
f1 (ppm)

^{19}F NMR Spectra

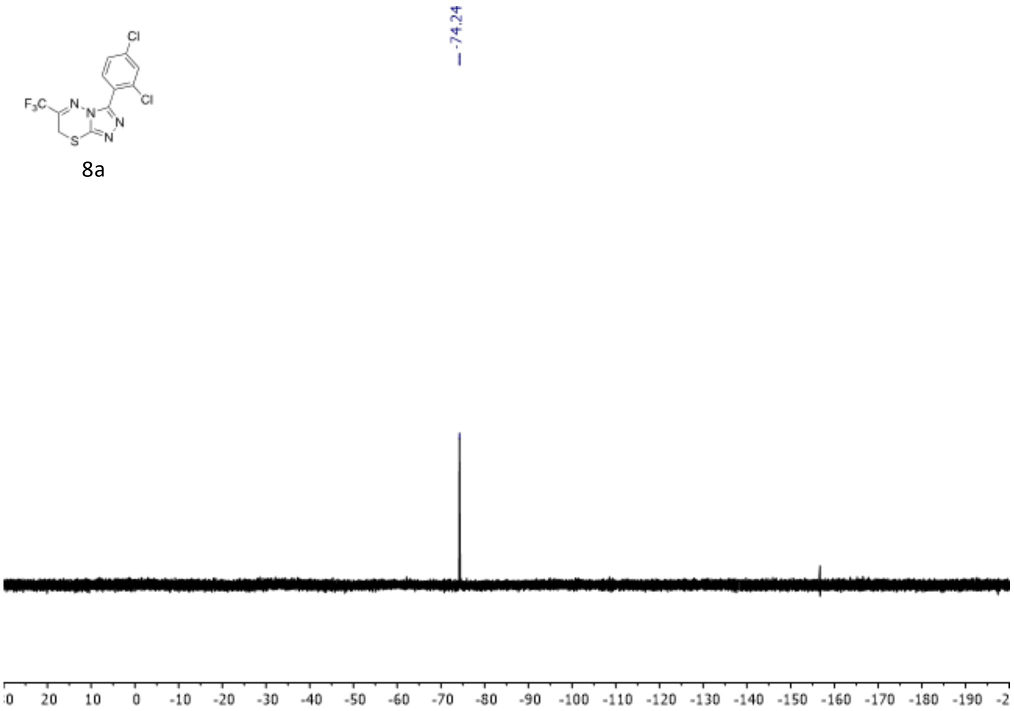

8a

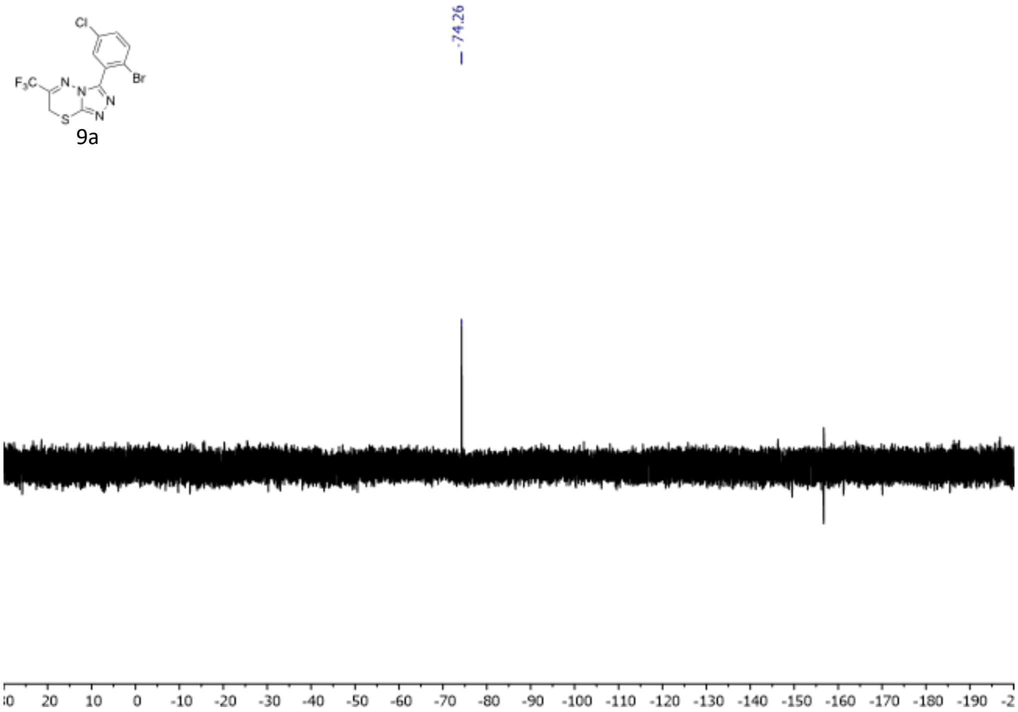

9a

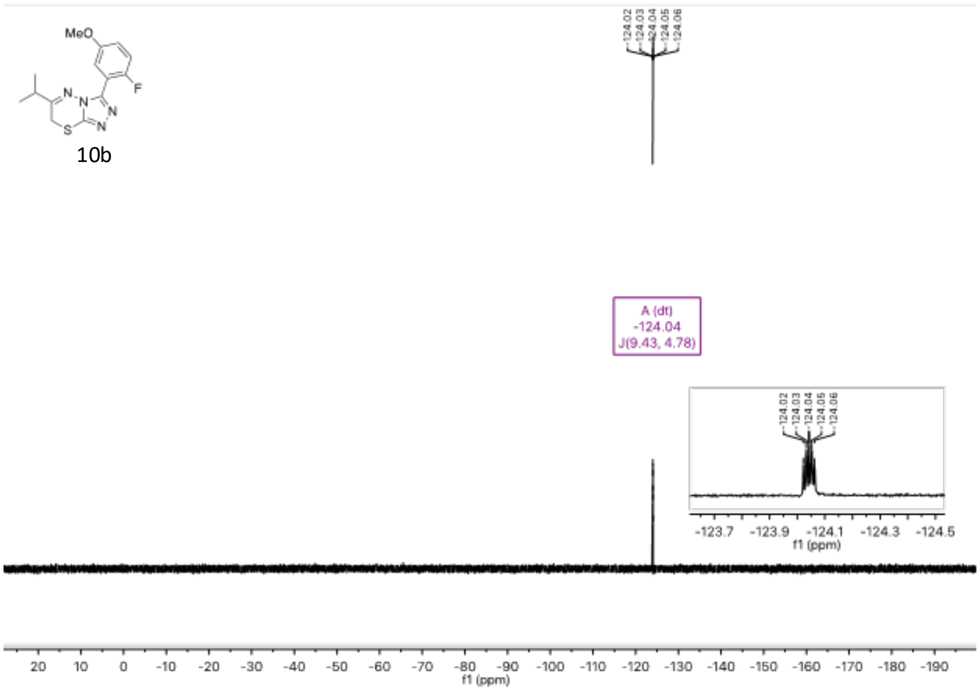
MeO
F
S
10b
-124.02
-124.03
-124.04
-124.05
-124.06
A (dt)
-124.04
J(9.43, 4.78)
-123.7 -123.9 -124.1 -124.3 -124.5
f1 (ppm)
20 10 0 -10 -20 -30 -40 -50 -60 -70 -80 -90 -100 -110 -120 -130 -140 -150 -160 -170 -180 -190
f1 (ppm)
